# Apache is a neuronal player in autophagy required for retrograde axonal transport of autophagosomes

**DOI:** 10.1007/s00018-024-05441-7

**Published:** 2024-10-05

**Authors:** Barbara Parisi, Alessandro Esposito, Enrico Castroflorio, Mattia Bramini, Sara Pepe, Antonella Marte, Fabrizia C. Guarnieri, Flavia Valtorta, Pietro Baldelli, Fabio Benfenati, Anna Fassio, Silvia Giovedì

**Affiliations:** 1 Dipartimento di Medicina Sperimentale, Università degli Studi di Genova, Genova, Italia; 2 Center for Synaptic Neuroscience and Technology, Italian Institute of Technology, Genoa, Italy; 3 IRCCS, Ospedale Policlinico San Martino, Genova, Italia; 4 Division of Neuroscience, IRCCS San Raffaele Scientific Institute, Milan, Italy; 5 Vita-Salute San Raffaele University, Milan, Italy; 6 Present Affiliation: IRCSS, Ospedale Policlinico San Martino, Genova, Italy; 7 Present Affiliation: Institute of Neuroscience, National Research Council (CNR), Vedano al Lambro, Italy; 8 Present Affiliation: Department of Cell Biology, Universidad de Granada, Granada, Spain; 9 Department of Experimental Medicine, University of Genoa, Viale Benedetto XV, 3, Genova, 16122 Italy

**Keywords:** Amphisome, Retrograde trafficking, Synapse, mTOR, Torin1, LC3, TrkB, AP-2

## Abstract

Neurons are dependent on efficient quality control mechanisms to maintain cellular homeostasis and function due to their polarization and long-life span. Autophagy is a lysosomal degradative pathway that provides nutrients during starvation and recycles damaged and/or aged proteins and organelles. In neurons, autophagosomes constitutively form in distal axons and at synapses and are trafficked retrogradely to the cell soma to fuse with lysosomes for cargo degradation. How the neuronal autophagy pathway is organized and controlled remains poorly understood. Several presynaptic endocytic proteins have been shown to regulate both synaptic vesicle recycling and autophagy. Here, by combining electron, fluorescence, and live imaging microscopy with biochemical analysis, we show that the neuron-specific protein APache, a presynaptic AP-2 interactor, functions in neurons as an important player in the autophagy process, regulating the retrograde transport of autophagosomes. We found that APache colocalizes and co-traffics with autophagosomes in primary cortical neurons and that induction of autophagy by mTOR inhibition increases LC3 and APache protein levels at synaptic boutons. APache silencing causes a blockade of autophagic flux preventing the clearance of p62/SQSTM1, leading to a severe accumulation of autophagosomes and amphisomes at synaptic terminals and along neurites due to defective retrograde transport of TrkB-containing signaling amphisomes along the axons. Together, our data identify APache as a regulator of the autophagic cycle, potentially in cooperation with AP-2, and hypothesize that its dysfunctions contribute to the early synaptic impairments in neurodegenerative conditions associated with impaired autophagy.

**Supplementary Information:**

The online version contains supplementary material available at 10.1007/s00018-024-05441-7.

## Introduction

Neurons are highly polarized post-mitotic cells that communicate through synaptic transmission. To sustain proper synaptic activity, neurons require high energy expenditure, constant protein turnover and removal of damaged organelles from synaptic sites [1, 2]. Diverse quality-control mechanisms are in place in all cells to monitor for the integrity of proteins and organelles and to determine their degradation. These include autophagy and the endo-lysosomal pathway, which are receiving increasing attention in the maintenance of neuronal and synaptic proteome homeostasis [3, 4].

Macroautophagy (hereafter autophagy) is a major degradative pathway that implies the bulk or selective engulfment of material in a double-membrane organelle called autophagosome, which eventually fuses either with lysosomes, generating an autolysosome, or initially with late endosomes to form amphisomes that later fuse with lysosomes to achieve cargo degradation. Autophagosome biogenesis requires, among other key autophagy factors, ATG5 and ATG3, involved in the elongation of the isolated membrane and in the lipid conjugation of the autophagic marker microtubule-associated protein 1 light chain 3 (LC3; [5, 6]). In neurons, this highly conserved process requires very specific regulatory mechanisms, given the extreme specialization of this type of cell. While core components of the autophagic machinery, extensively studied in non-neuronal cell lines, are conserved in neurons, neuron-specific regulators start to be identified [7, 8].

Autophagosomes are particularly abundant at distal axons and presynaptic terminals [9]. Following fusion with late endosomes, the amphisomes, containing both the late endosome marker Rab7 and LC3, begin a coordinated process of maturation and retrograde transport along microtubules powered by the motor protein dynein toward the neuronal cell body, where the bulk of degradative lysosomes are located [10, 11]. At synapses, many proteins involved in the recycling of synaptic vesicles (SVs) have been found to play a role in the biogenesis of presynaptic autophagosomes and in their retrograde transport [12]. Indeed, autophagosome formation is driven by presynaptic endocytic factors such as synaptojanin and endophilinA [13–16], while it is repressed by the active zone scaffolding protein Bassoon [17]. The autophagic protein ATG9A, a lipid scramblase involved in phagophore formation and extension, is present on SVs and its sorting to the nascent autophagosomal membrane depends on the clathrin adaptor protein complex AP-2 and on the active zone protein Clarinet [18, 19]. AP-2 has also been implicated in the autophagic delivery of ATG16L1, a protein involved in autophagosome membrane expansion [20], as well as in the retrograde trafficking of TrkB-containing signaling amphisomes through the interaction with the autophagic protein LC3 and the dynein motor adaptor dynactin [21], unveiling an additional non-canonical role in autophagic process.

Loss or impairment of autophagy can lead to defects in neuronal development, altering processes such as axonal growth, synaptogenesis and synaptic function, thus supporting a key role of the process in neuronal physiology and survival [21–24].

We have recently shown that the neuron-specific protein APache interacts with AP-2 on clathrin-coated vesicles and is implicated in clathrin-mediated endocytosis of SVs at presynaptic terminals [25]. Given that key molecular components are shared by autophagy and the SV recycling machinery, we hypothesized that APache might contribute to the autophagy pathway. Indeed, we found that APache expression is coordinated with autophagy induction through the mammalian target of rapamycin (mTOR) inhibition at synaptic level. APache silencing in neurons leads to defective p62/SQSTM1 protein degradation and accumulation of autophagic vacuoles (AVs) that are not properly retro-transported to the cell soma and cleared. The results indicate that APache, acting as part of a protein complex comprising the adaptor AP-2 and the dynein cofactor dynactin, is a novel important player regulating autophagic flux and the retrograde transport of autophagosomes in neurons.

## Results

### APache is induced by autophagy stimulation and colocalizes with LC3 on autophagosomes

**Fig. 1:**
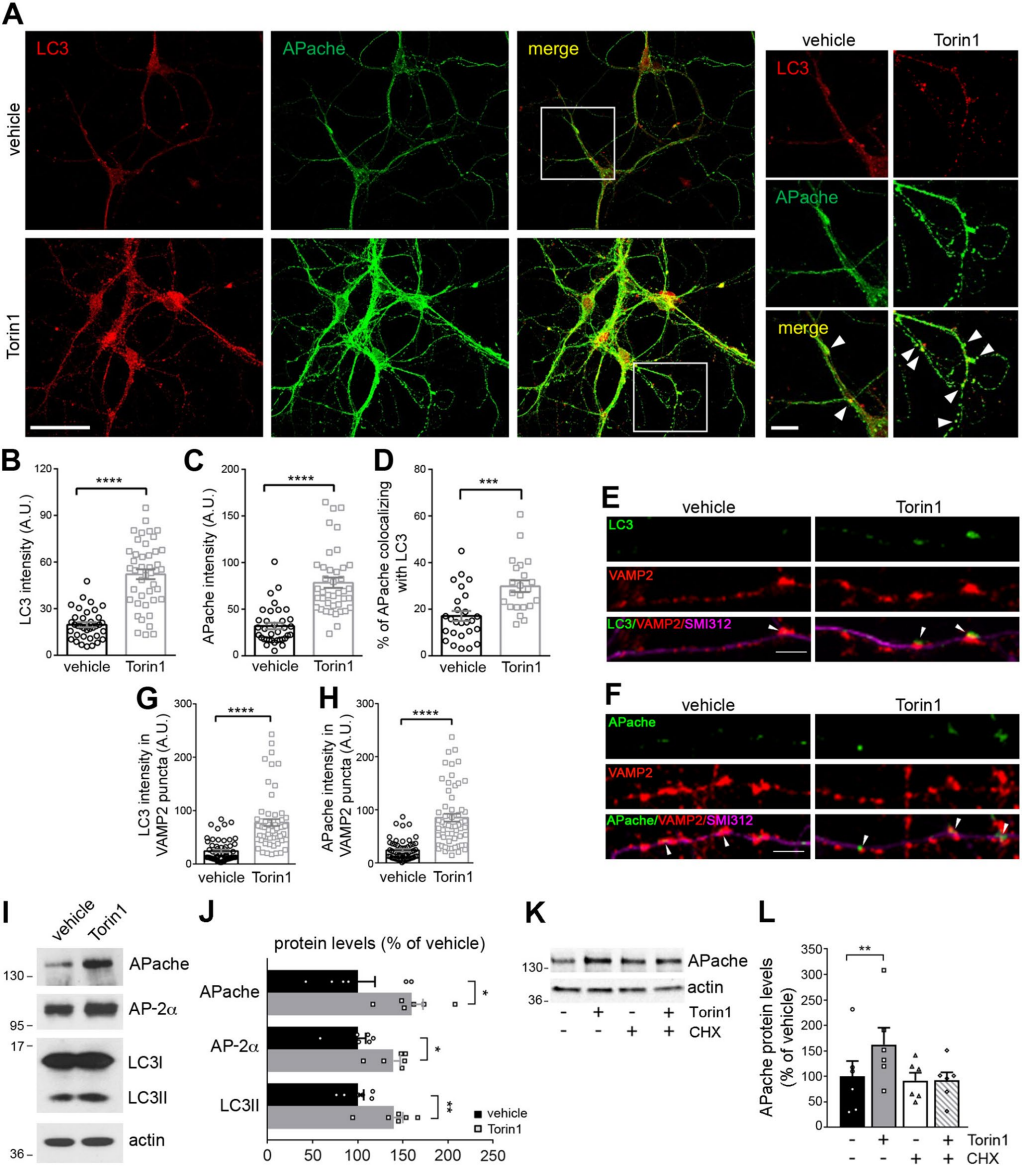
Increased APache protein levels colocalize with LC3 after autophagy induction by mTOR inhibition. (**A**) Representative confocal images of cortical neurons treated with either DMSO (vehicle) or the mTOR inhibitor Torin1 (250 nM, 4 h) at 17 DIV and stained for LC3 (red) and APache (green). White boxes indicate panels magnified to the right. Arrowheads in the magnified inserts denote points of co-localization between LC3-positive structures and APache. Scale bars: 20 μm, 5 μm (inserts). (**B**-**D**) Quantification of fluorescence intensity values of LC3 (**B**, vehicle: 19.68 ± 1.60; Torin1: 52.13 ± 3.15), APache (**C**, vehicle: 31.96 ± 3.40; Torin1: 78.31 ± 5.53) and of the percent of APache/LC3 co-localization based on Manders’ coefficient (**D**, vehicle: 17.10 ± 2.13%; Torin1: 29.85 ± 2.50%, *n* = 22–44 neurons, from 3 independent preparations) in vehicle- and Torin1-treated neurons. (**E**,** F**) Representative confocal images of cortical neurons treated with either vehicle or Torin1 and triple stained for VAMP2 to identify synaptic boutons (red), SMI312 to label axons (magenta) and either LC3 (green, **E**) or APache (green, **F**) at 17 DIV. Arrowheads in the merge panels indicate synaptic boutons positive for LC3 or APache. Scale bar, 5 μm. (**G**,** H)** Quantification of LC3 (**G**) or APache (**H**) fluorescence intensity values at VAMP2-positive puncta in vehicle- and Torin1-treated synapses (G, LC3 vehicle: 19.50 ± 1.95; LC3 Torin1: 65.78 ± 4.99; H, APache vehicle: 18.99 ± 1.81; APache Torin1: 69.56 ± 5.94, *n* = 82–89 synapses, from 3 independent preparations). A.U. = arbitrary units of fluorescence intensity. (**I**,** J**) Representative blots (**I**) and quantitative analysis (**J**) of lysates of cortical neurons treated at 17 DIV with either DMSO (vehicle) or Torin1 (250 nM, 4 h). Treated neurons show increased expression levels of the active autophagic marker LC3II, APache and AP-2α. Actin was used as loading control. Protein levels in treated neurons are expressed in percent of control neurons. (APache Torin1: 160 ± 12.3%; AP-2α Torin1: 139.6 ± 7.58%; LC3II Torin1: 138.9 ± 10.09%, from *n* = 6 independent preparations). (**K**,** L**) Representative blots (**K**) and quantitative analysis (**L**) of the expression level of APache in lysates from cultured cortical neurons treated at 17 DIV with either DMSO/water (vehicle), Torin1 (250 nM, 4 h) and/or cycloheximide (CHX, 5 µg/ml, 4 h). Actin was used as loading control. APache protein levels are expressed in percent of control neurons (Torin1: 162.4 ± 33.27%; CHX: 91.91 ± 15.4%; Torin1/CHX: 92.01 ± 16.12%, from *n* = 6 independent preparations). All graphs show means ± SEM. *p < 0.05; **p < 0.01; ***p < 0.001; ****p < 0.0001, unpaired Student’s *t*-test with Welch’s correction (**B**, **D**, **J**), Mann Whitney’s *U*-test (**C**, **G**, **H**); **p < 0.01, one-way ANOVA/Bonferroni’s tests (**L**). See also Figure S1

To address the functional role of APache in autophagy, we initially examined whether the induction of autophagy by treatment with the mTOR inhibitor Torin1 (250 nM, 4 h) affected the levels of APache in mature cortical neurons. mTOR is the major negative regulator of autophagy, sensing nutrient deprivation and other forms of stress; however, how it regulates neuronal autophagy is still incompletely understood [10]. Immunocytochemistry revealed a significant increase in LC3 immunoreactivity in Torin1-treated neurons compared to vehicle-treated cells (Fig. 1A, B), consistent with the known induction of autophagy by Torin1 in neurons [26]. Interestingly, also APache immunoreactivity and colocalization with LC3 significantly increased in Torin1-treated neurons compared to controls (Fig. 1A, C, D), particularly at synaptic sites identified by immunostaining with the synaptic marker VAMP2 (Fig. 1E-H, Fig. S1). These results confirm the accumulation of newly formed autophagosomes at synapses after mTOR-dependent stimulation of autophagy [27] and uncover the concomitant APache protein induction at synaptic level and its recruitment to LC3-positive structures.

**Fig. 2:**
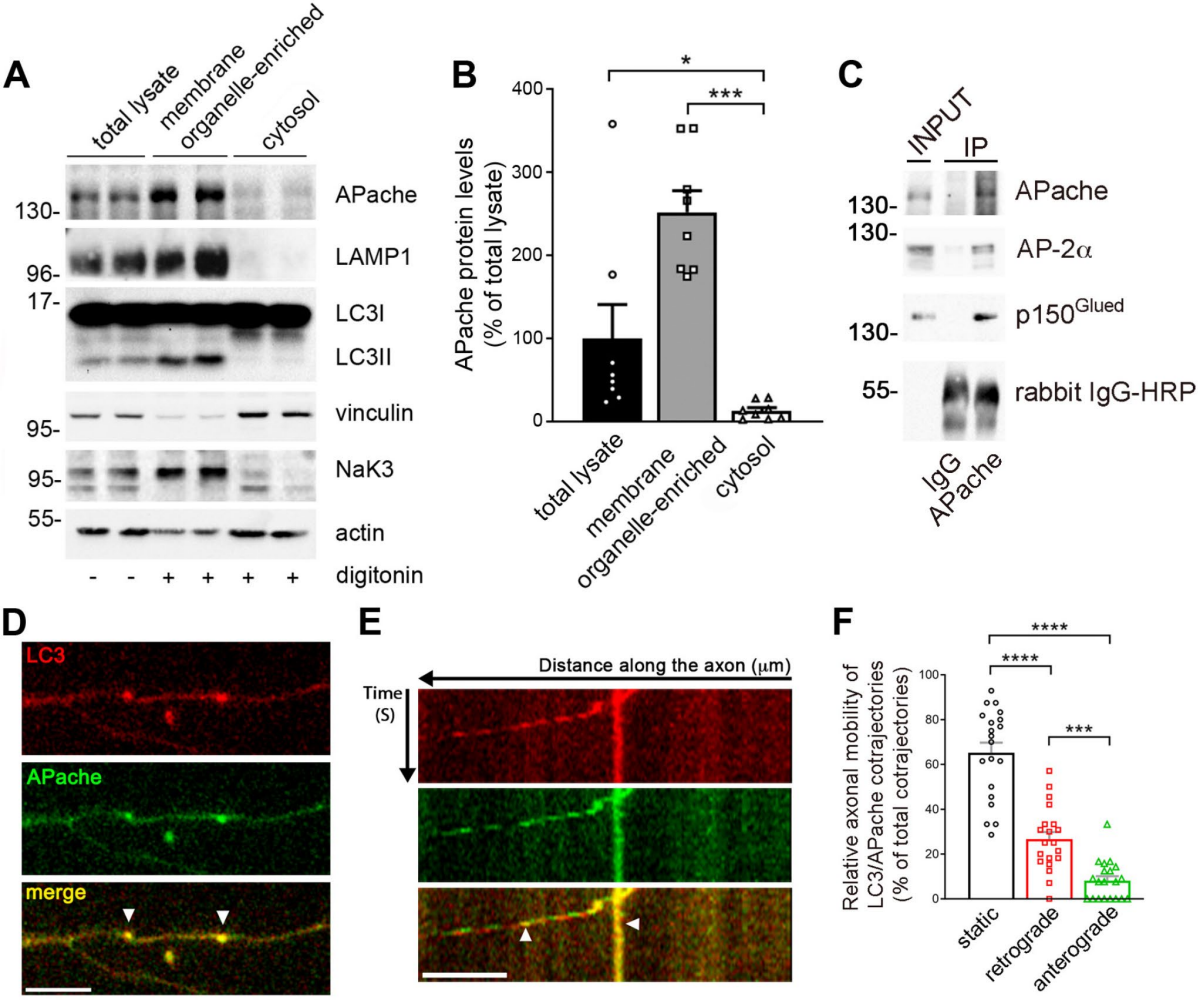
APache forms a complex with AP-2 and dynactin and co-traffics with LC3 in cultured cortical neurons. (**A**) Representative blots of total lysates and plasma membrane/organelle-enriched and cytosolic fractions obtained with 0.02% digitonin treatment of cultured cortical neurons at 17 DIV, run in duplicate. Autophagic LC3II and late endosomal/lysosomal LAMP1 proteins were used to confirm the enrichment in AVs (autophagosomes/amphisomes/autolysosomes) in the organelle-enriched fraction. Cytoplasmic vinculin was adopted to evaluate potential cytosolic contaminations in the organellar fraction and for cytosolic loading control, Na^+^/K^+^ ATPase α-3 (NaK3) was used for membrane loading control, and actin for total loading control. Each lane contains equal protein loading. (**B**) Quantitative analysis of APache shows an increased protein level in the vacuolar-enriched fraction. Protein levels are expressed in percent of total lysates (membrane/organelle-enriched: 251.5 ± 25.85%; cytosol: 12.78 ± 3.73%, from *n* = 6 independent preparations). (**C**) Mouse brain extracts were subjected to immunoprecipitation (IP) assays with anti-APache polyclonal antibodies or control IgGs. Equal aliquots (2% of total) of the starting material (INPUT) together with the IP samples (20% of total for APache and IgG and 80% of total for AP-2α and dynactin) were subjected to immunoblotting with the indicated antibodies. APache co-immunoprecipitated dynactin and AP-2. The IPs were performed twice with similar results. (**D**,** E**) Mouse cortical neurons were co-transfected at 11 DIV with EGFP-APache and RFP-LC3 and analyzed at 14 DIV. Representative epifluorescence images and corresponding kymographs showing the colocalization (**D**) and co-transport (**E**) of EGFP-APache with RFP-LC3-labelled autophagosomes (arrowheads). Scale bar, 5 μm. (**F**) Relative axonal mobility of LC3/APache co-trajectories expressed in percent of the total number of co-trajectories (static: 65.23 ± 4.48%; retrograde: 26.62 ± 3.22%; anterograde: 8.15 ± 1.95%, *n* = 226 co-trajectories from *n* = 20 neurons, from *n* = 3 independent preparations). All graphs show means ± SEM. *p < 0.005; ***p < 0.001, Kruskal-Wallis’s ANOVA/Dunn’s tests (**B**); ***p < 0.001; ****p < 0.0001, one-way ANOVA (**F**)

Western blot analysis in total cell lysates after autophagy stimulation corroborated the increased protein expression of autophagosome-associated lipidated LC3 (LC3II), α subunit of the heterotetrameric adaptor protein AP-2 and APache (Fig. 1I, J). APache accumulation was prevented upon inhibition of protein synthesis by cycloheximide (5 µg/ml) (Fig. 1K, L), indicating that APache upregulation is due to induction of protein synthesis by stimulation of autophagy and not to defective degradation. The localization of APache in neurons and its association with autophagosomes was analyzed by performing digitonin-based subcellular fractionation experiments for effectively separating cytosolic from plasma membrane and organelle proteins. A comparison of the total lysate and cytosolic fractions indicated that APache was enriched in the membranous compartment associated with the vacuolar-enriched fraction positive for LC3II and LAMP1 (Fig. 2A, B). Moreover, in line with previous results [25], we found endogenous APache to co-immunoprecipitate with the p150^Glued^ subunits of dynactin, cofactor for the retrograde motor protein dynein, and AP-2α (Fig. 2C), suggesting that the three proteins may act as part of a complex that mediates the retrograde transport of autophagosomes in neurons [21]. Finally, to investigate whether APache co-transports with LC3-positive structures, we monitored the dynamics of fluorescence-tagged proteins by live imaging of primary cortical neurons in culture. This analysis revealed a close colocalization and co-trafficking of LC3-RFP with APache-GFP, whose mobility was predominantly retrograde, consistent with dynein-driven behavior (Fig. 2D-F). Altogether, the data suggest that induction of autophagy in neurons by mTOR inhibition promotes APache upregulation also at the synaptic level and its colocalization and co-trafficking with LC3 on autophagosomes.

### APache silencing causes an aberrant accumulation of autophagic vacuoles also at synaptic level in cortical neurons

**Fig. 3:**
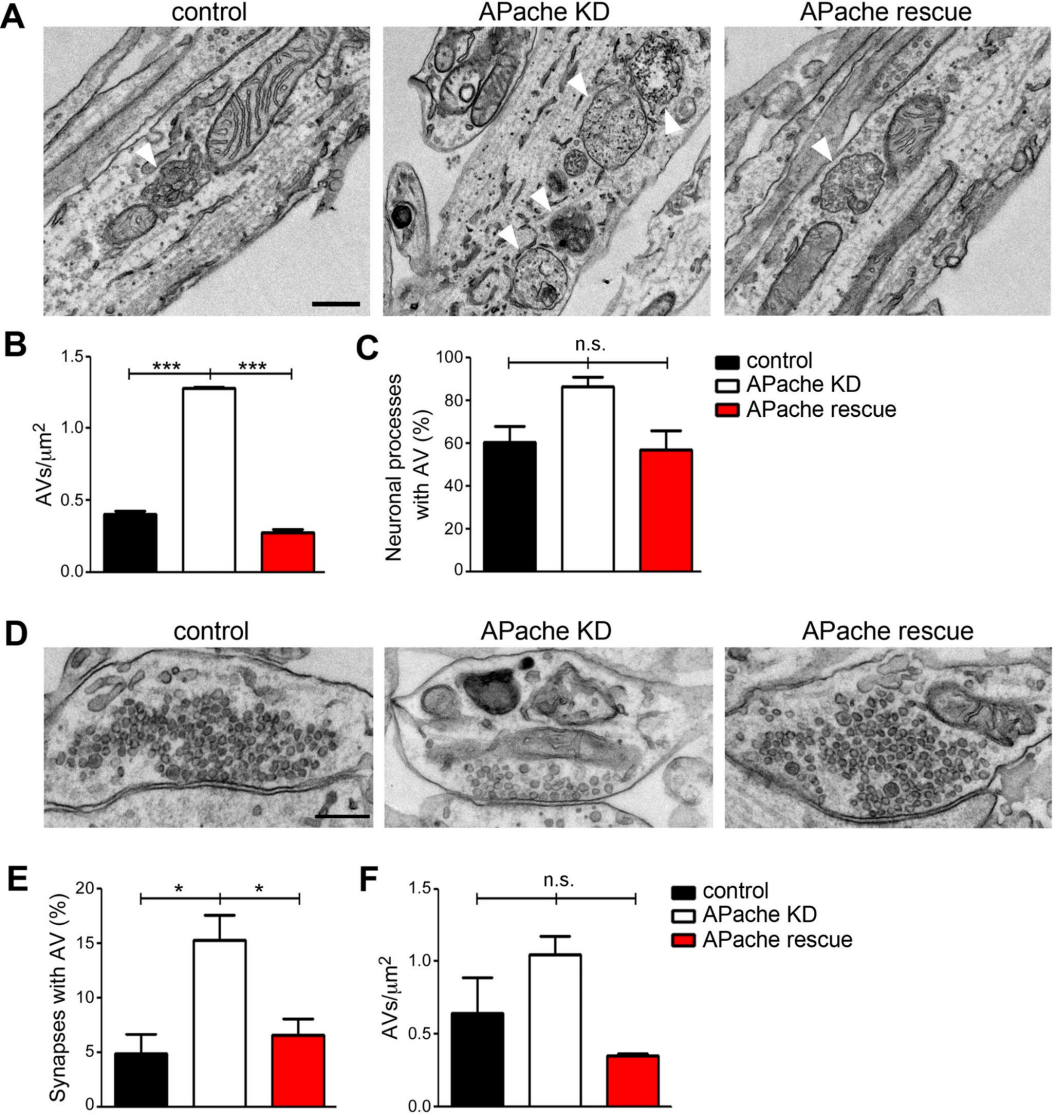
APache silencing results in an aberrant accumulation of autophagic vacuoles in cortical neurons. (**A**) Representative electron micrographs of neurites from cultured cortical neurons transduced at 12 DIV with lentiviral vectors coding for either control mCherry-shRNA (control), APache mCherry-shRNA (APache KD) or rescued by co-transduction with shRNA-resistant EGFP-APache (APache rescue) and processed at 17 DIV. White arrowheads indicate autophagic vacuoles (AVs). Scale bar, 200 nm. (**B**,** C**) Quantification of the AV density (**B**) and the percent of neuronal processes containing AVs (**C**) in control (black bars), APache KD (white bars) and APache rescue (red bars) neurons (*n* = 54 images per genotype, from 3 independent preparations). (B, control: 0.4 ± 0.022; APache KD: 1.28 ± 0.008; APache rescue: 0.272 ± 0.025; C, control: 60.248 ± 7.687%; APache KD: 86.364 ± 4.545%; APache rescue: 56.818 ± 9.185%). (**D**) Representative electron micrographs of presynaptic terminals from control, APache KD or APache rescue cortical neurons. Scale bar, 200 nm. (**E**,** F**) Quantification of the percent of synapses containing AVs (**E**) and the AV density (**F**) in control, APache KD and APache rescue neurons (*n* = 114 control synapses, *n* = 100 APache KD synapses and *n* = 50 APache rescue synapses, from 4 independent preparations). (E, control: 4.897 ± 1.799%; APache KD: 15.278 ± 2.240%; APache rescue: 6.6 ± 1.5%; F, control: 0.641 ± 0.25; APache KD: 1.05 ± 0.128; APache rescue: 0.349 ± 0.015). All graphs show means ± SEM. *p < 0.05; ***p < 0.001; n.s.= non-significant, one-way ANOVA/Bonferroni’s tests

To test whether APache plays a role in neuronal autophagy, we acutely silenced its expression in cultured primary mouse cortical neurons by RNAi [25] and examined cell ultrastructure by transmission electron microscopy (TEM). Along neurites, APache knockdown (KD) neurons displayed a marked and highly significant accumulation of AVs compared to control neurons with a trend for an increased percentage of AV-containing neuronal processes (Fig. 3A-C). At the synaptic level, in addition to the previously described reduced SV density [25], APache KD brought about a significant increase of the percentage of AV-containing synapses, associated with a trend toward an increase in AV density (Fig. 3D-F). Notably, the ultrastructural effects of APache silencing were reversible: the silencing phenotype was completely rescued by co-transduction of neurons with an shRNA-resistant APache cDNA (Fig. 3A-F), effective in overexpressing EGFP-APache in silenced neurons [25].

**Fig. 4:**
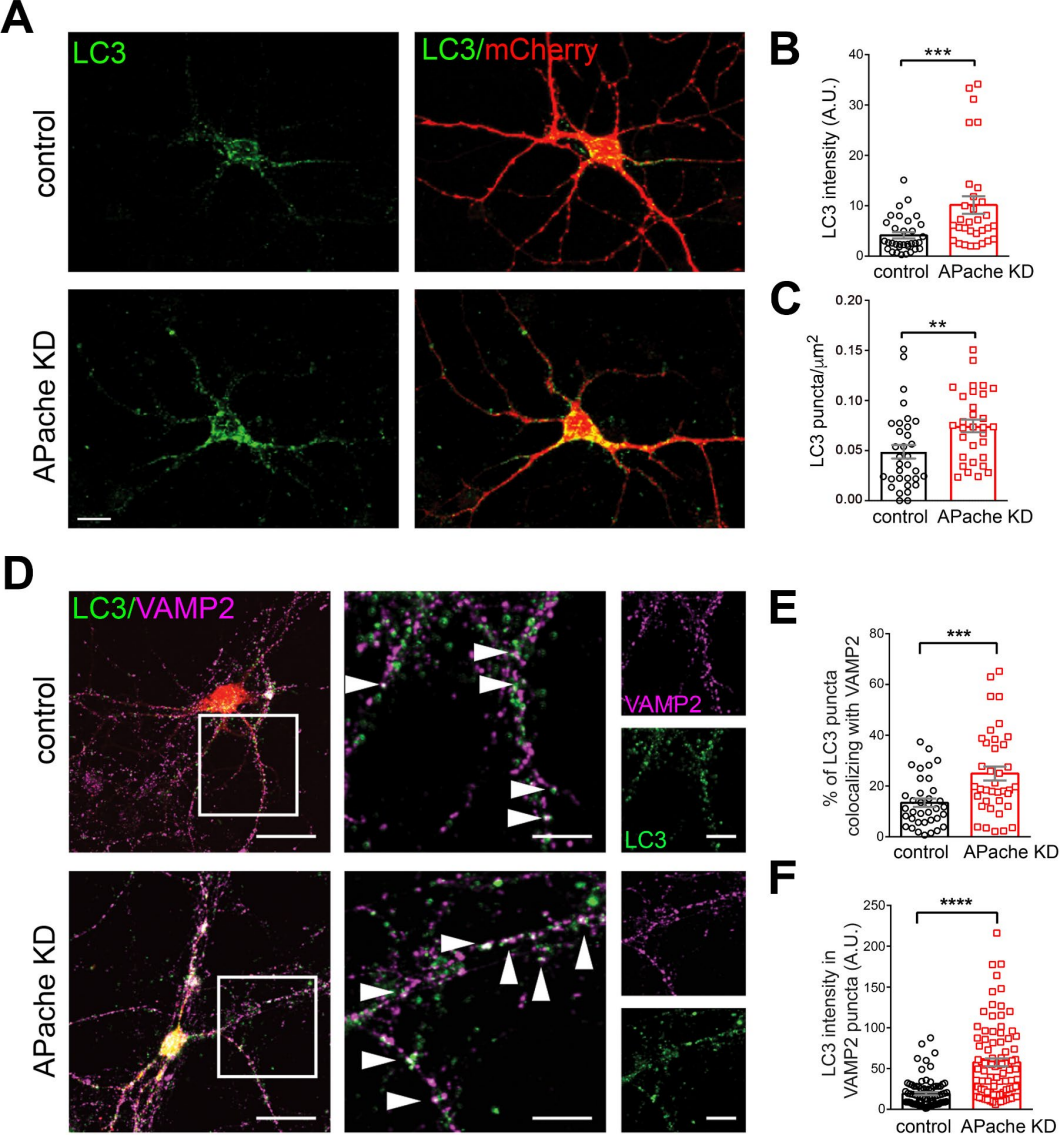
APache silencing increases LC3-positive structures in cortical neurons. (**A**) Representative confocal images of cortical neurons transduced at 12 DIV with lentiviral vectors coding for either control mCherry-shRNA (control) or APache mCherry-shRNA (APache KD) and stained for LC3 (green) at 17 DIV. Scale bar, 10 μm. (**B**) Quantification of LC3 fluorescence intensity in control and APache KD neurons (control: 4.14 ± 0.61; APache KD: 10.18 ± 1.73, *n* = 31–33 neurons, from 3 independent preparations). (**C**) Quantification of LC3-puncta density in control and APache KD neurons (control: 0.048 ± 0.007; APache KD: 0.074 ± 0.006, *n* = 31–33 neurons, from 3 independent preparations). (**D**) Representative confocal images of either control or APache KD cortical neurons double stained for LC3 (green) and VAMP2 (magenta) to identify synaptic boutons. White boxes indicate neurites shown on the right at higher magnification. Arrowheads in the magnified inserts denote synaptic boutons positive for LC3-positive structures. Scale bars: 20 μm, 10 μm (inserts). (**E**) Quantification of the percent of LC3 puncta colocalizing with VAMP2 in control and APache KD neurons based on Manders’ coefficient (control: 13.42 ± 1.63%; APache KD: 24.91 ± 2.72%, *n* = 36–38 neurons, from 3 independent preparations). (**F**) Quantification of LC3 fluorescence intensity values at VAMP2-positive puncta in control and APache KD neurons (control: 19.2 ± 1.84; APache KD: 57.61 ± 4.83, *n* = 87–88 synapses, from 3 independent preparations). A.U. = arbitrary units of fluorescence intensity. All graphs show means ± SEM. **p < 0.01; ***p < 0.001, Mann-Whitney’s *U*-test (**B**, **C**), ***p < 0.001; ****p < 0.0001, unpaired Student’s *t*-test with Welch’s correction (**E**, **F**). See also Figure S2

**Fig. 5:**
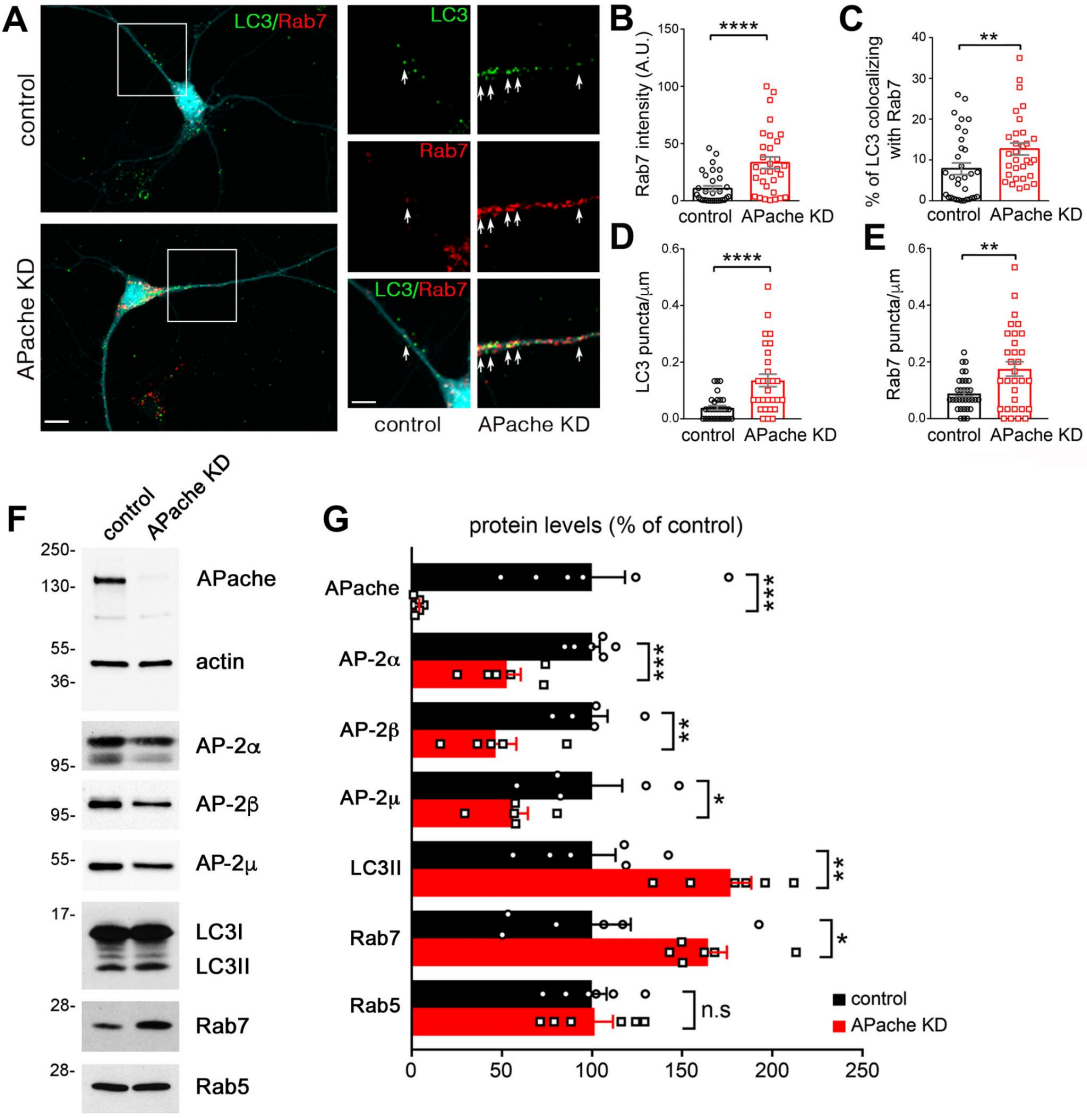
Accumulation of amphisomes in cortical neurons after APache silencing. (**A**) Representative confocal images of cortical neurons transduced at 12 DIV with lentiviral vectors coding for either control mCherry-shRNA (control) or APache mCherry-shRNA (APache KD) and double stained for LC3 (green) and Rab7 (red) at 17 DIV. Channels were pseudocolor-coded to better illustrate the co-localization (mCherry-shRNA displayed in cyan). White boxes indicate proximal neurites shown on the right at higher magnification. Arrows in the magnified inserts indicate points of co-localization between LC3 and Rab7. Scale bars: 10 μm, 5 μm (inserts). (**B**) Quantification of Rab7 fluorescence intensity in control and APache KD neurons (control: 10.55 ± 2.40, APache KD: 33.24 ± 5.09, *n* = 31 neurons from 3 independent preparations). A.U. = arbitrary units of fluorescence intensity. (**C**) Quantification of the percent of LC3 puncta colocalizing with Rab7 in control and APache KD neurons based on Manders’ coefficient (control: 7.84 ± 1.42%, APache KD: 12.68 ± 1.46%, *n* = 31–33 neurons from 3 independent preparations). (**D**,** E**) Quantification of the number of LC3- (**D**) and Rab7- (**E**) positive puncta counted on 30-µm axonal branches starting from the cell body in control and APache KD neurons (LC3 control: 0.038 ± 0.008, LC3 APache KD: 0.135 ± 0.022, Rab7 control: 0.088 ± 0.010, Rab7 APache KD: 0.175 ± 0.025, *n* = 29–32 neurons, from 3 independent preparations). (**F**,** G**) Representative blots (**F**) and quantitative analysis (**G**) of lysates from control and APache KD neurons at 17 DIV. Compared to control neurons, APache KD neurons show increased expression levels of LC3II and Rab7 (late endosomal marker) and decreased expression levels of AP-2α, β and µ subunits. No significant difference in Rab5 (early endosomal marker) expression has been observed. Actin, shown with APache on the same blot, was used as loading control. Protein levels in APache KD neurons are expressed in percent of control neurons (APache: 3.26 ± 0.89%; AP-2α: 52.74 ± 7.7%; AP-2β: 46.12 ± 9.13%; AP-2µ: 58.19 ± 6.03%; LC3II: 176.8 ± 11.6%; Rab7: 164.5 ± 10.45%; Rab5: 101.4 ± 10.16%, from *n* = 5–6 independent preparations). All graphs show means ± SEM. **p < 0.01; ****p < 0.0001, Mann-Whitney’s *U*-test (**B**-**D**); *p < 0.05; **p < 0.01; ***p < 0.001, unpaired Student’s *t*-test (**E**, **G**)

Consistent with our TEM studies, analysis of APache KD neurons by fluorescence microscopy revealed an increased density and fluorescence intensity of LC3-puncta (Fig. 4A–C), and a higher percentage of synapses containing LC3-positive structures compared to control neurons (Fig. 4D–F), in the absence of significant changes in the density of *bona fide* presynaptic boutons (Fig. S2). These data suggest that APache silencing impairs autophagy causing an accumulation of autophagosomal vesicles also at the synaptic level. To characterize the nature of accumulating autophagosomes upon APache downregulation, we also investigated the expression and distribution of the late endosome marker Rab7. The accumulation of amphisomes, late-stage autophagosomes after fusion with late endosomes, in APache-silenced neurons was evidenced by the more intense immunoreactivity of Rab7 and its enhanced colocalization with LC3 compared to controls (Fig. 5A–C). Also, the number of LC3- and Rab7-positive puncta along neurites was increased in APache KD neurons (Fig. 5D, E). Moreover, immunoblotting analysis showed increased protein level of LC3II and Rab7 in APache KD neurons, without significant alterations of the early endosome marker Rab5 (Fig. 5F, G), confirming an accumulation of amphisomes in APache-silenced neurons. In agreement with previous results [25], the protein levels of the α-subunit of AP-2, but also of the β and µ subunits, were significantly reduced in APache-downregulated cells (Fig. 5F, G). Collectively, these findings demonstrate that the absence of APache in cortical neurons results in a strong accumulation of autophagic structures, mainly amphisomes, also at the presynaptic level.

### APache silencing results in the blockade of the autophagic flux

Since accumulation of autophagosomes in APache KD neurons could also result from increased autophagic activation, we investigated the protein levels of total and phosphorylated mTOR and ULK1 (unc-51-like kinase 1), as well as the levels of the early-core autophagy proteins ATG3 and ATG5 by immunoblotting. ULK1 is the key upstream autophagic modulator involved in autophagosome biogenesis, which is inhibited by mTOR-dependent phosphorylation at residue Ser757 [28]. No protein level alterations were detected in APache KD neurons compared to controls (Fig. S3), suggesting that the accumulation of autophagosomes in the absence of APache is not a result of reduced mTOR activity.

**Fig. 6:**
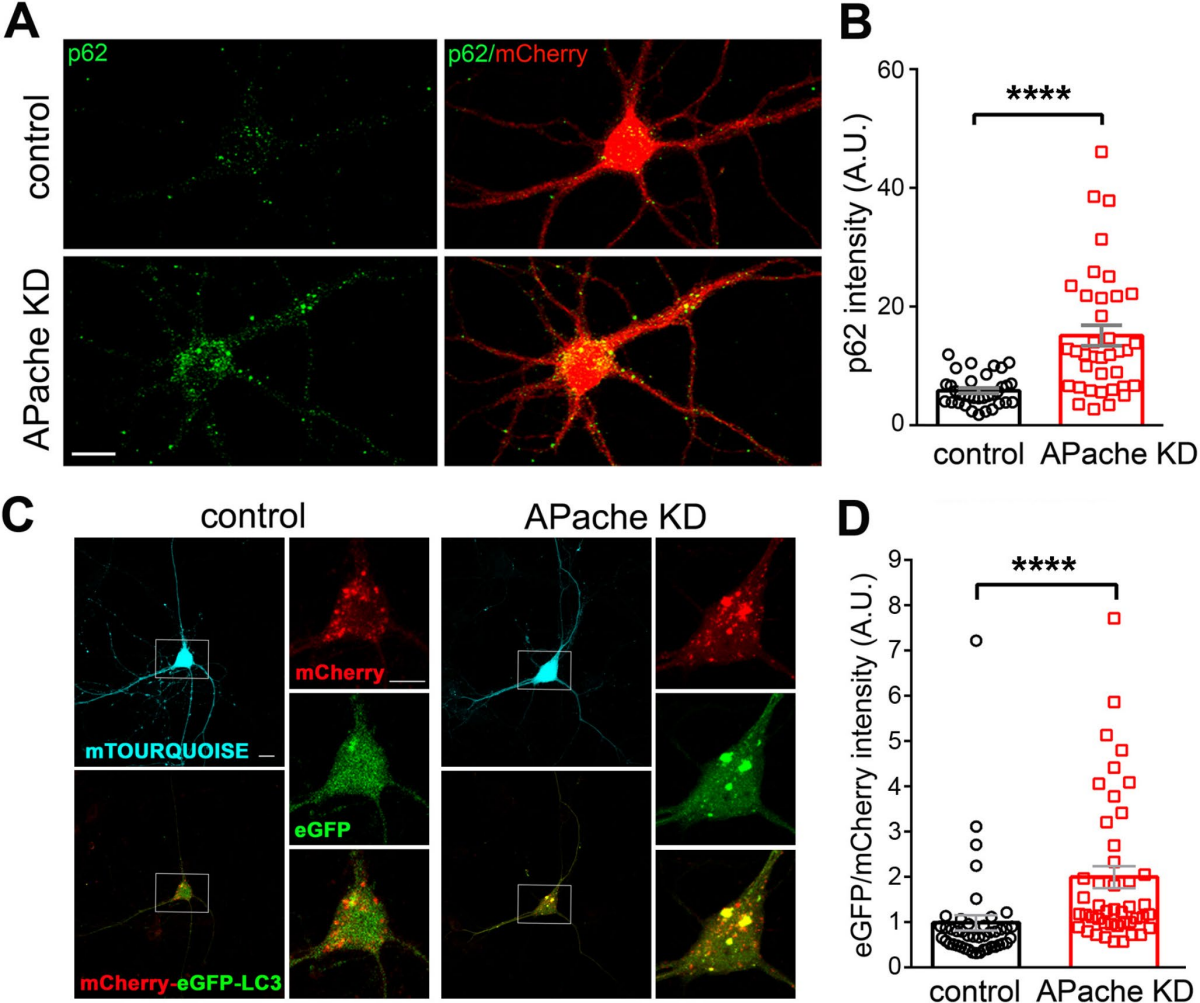
APache silencing blocks the autophagic flux in cortical neurons. (**A**) Representative confocal images of control or APache KD cortical neurons (mCherry-shRNA) stained for p62 (green) at 17 DIV. Scale bar, 10 μm. (**B**) Quantification of p62 fluorescence intensity values in control and APache KD neurons (control: 5.85 ± 0.45; APache KD: 15.17 ± 1.74, *n* = 34–37 neurons, from 3 independent preparations). A.U. = arbitrary units of fluorescence intensity. (**C**) Representative confocal images of mouse cortical neurons at 17 DIV co-transfected with vectors coding for either control mTourquoise-shRNA (control) or APache mTourquoise-shRNA (APache KD) and tandem mCherry-eGFP-tagged LC3, as a reporter of autolysosome formation, at 14 DIV. White boxes indicate the cell soma area shown on the right at higher magnification. Scale bars: 20 μm, 10 μm (inserts). (**D**) Mean eGFP/mCherry intensity ratio in control and APache KD neurons (control: 0.9861 ± 0.1692; APache KD: 1.997 ± 0.2414, *n* = 44–45 neurons, from 5 independent preparations). A.U. = arbitrary units of fluorescence intensity. All graphs show means ± SEM. ****p < 0.0001, Mann-Whitney’s *U*-test (**B**, **D**). See also Figure S3 and Figure S4

On the contrary, when we analyzed the levels of the autophagic receptor and degradative substrate p62/SQSTM1, which directly binds to LC3 and is degraded by autophagy [29, 30], in APache-depleted neurons by immunofluorescence, we observed a significant accumulation with respect to control neurons indicative of defective autophagic flux (Fig. 6A, B), providing evidence for a functional role of APache in the turnover of autophagic substrates. We next assessed the turnover of autophagosomes in APache KD neurons by using a tandem mCherry-eGFP-LC3, an established autophagic flux tracking system, as a reporter of autolysosome formation [31]. The significant increase in eGFP/mCherry intensity ratio observed in the cell body of APache KD neurons (Fig. 6C, D) demonstrates an accumulation of autophagosomes likely due to impaired fusion with lysosomes and not an increase in autophagy induction. The blockade of the autophagic flux caused by APache silencing indicates that APache is a new player in the regulation of neuronal autophagic process. To thoroughly investigate the molecular mechanisms governing the process, we analyzed the levels of LC3 and p62/SQSTM1 by fluorescence microscopy in APache-silenced neurons. Given the reduced expression levels of AP-2 in APache-depleted neurons, we overexpressed AP-2µ, the essential AP-2 subunit regulating retrograde autophagosome transport [21]. The fluorescence intensity values of both autophagic markers were significantly reduced by co-transfection of APache-KD neurons with mCherry-AP-2µ subunit (Fig. S4), making them comparable to those of non-silenced neurons (Figs. 4B and 6B), indicating that the overexpression of AP-2µ can reverse the blockade of the autophagic flux observed after APache loss. These results provide further evidence for a functional interaction of the two proteins as regulators of the autophagic cycle.

### APache controls the retrograde trafficking of TrkB/LC3-amphisomes in neurons

**Fig. 7:**
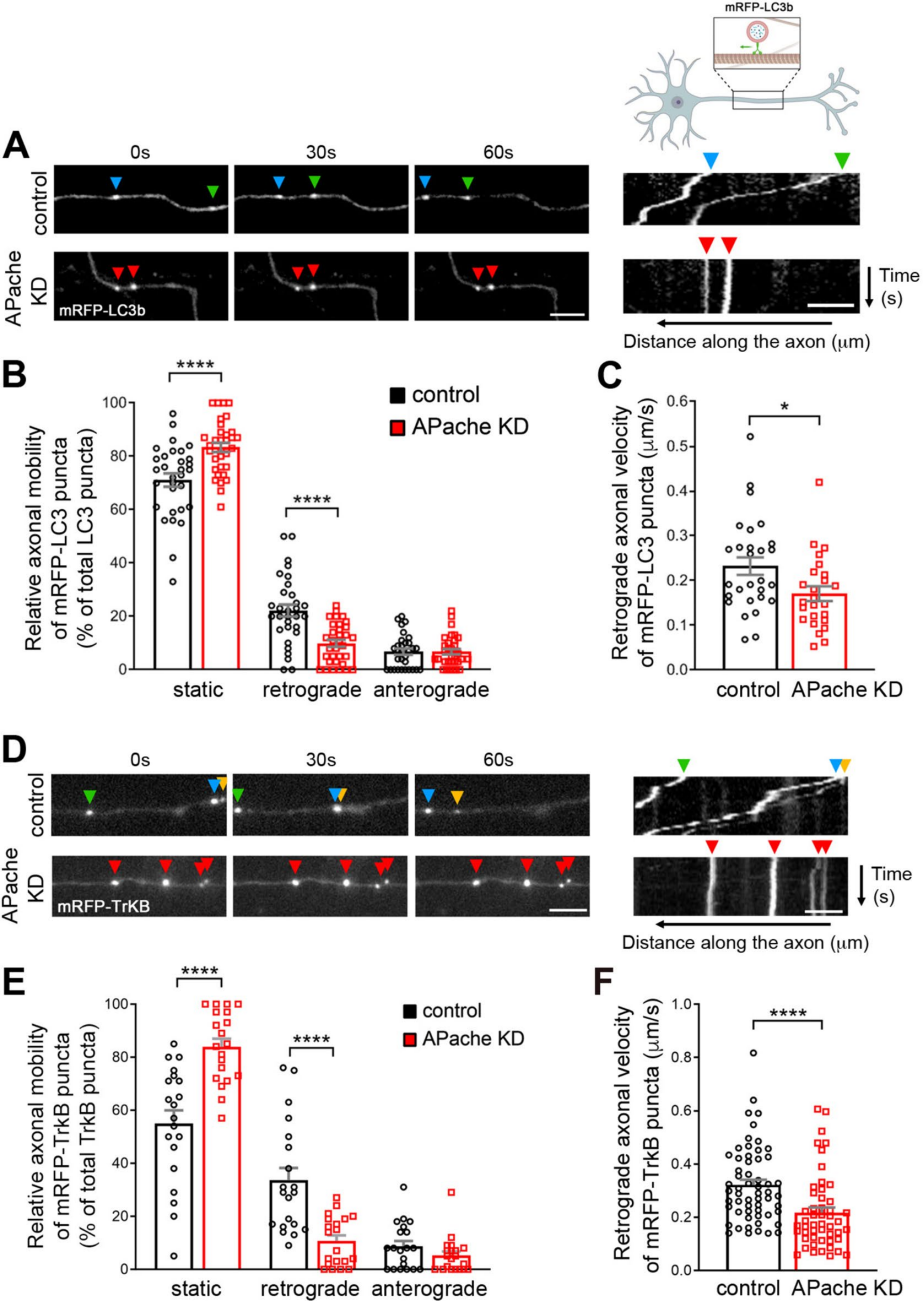
APache silencing alters the retrograde transport of LC3/TrkB-containing amphisomes in cortical neurons. (**A**) Time-lapse images of mRFP-LC3b positive puncta (arrowheads) along the axons of mouse cortical neurons at 14 DIV co-transfected with either control GFP-shRNA (control) or APache GFP-shRNA (APache KD) at 11 DIV. The corresponding representative kymographs of mRFP-LC3b puncta representing motion as displacement along the axon over time are shown on the right panels. Scale bar, 5 μm. (**B**) Relative axonal mobility of mRFP-LC3b puncta in control and APache KD neurons expressed in percent of total LC3 puncta. Deletion of APache significantly decreases the percentage of retrograde moving autophagosomes and concurrently increases that of stationary ones, while leaving unaltered the number of anterogradely moving autophagomes (control static: 71.10 ± 2.47%; APache KD static: 83.33 ± 1.76%; control retrograde: 22.19 ± 2.29%; APache KD retrograde: 9.94 ± 1.30%; control anterograde: 6.77 ± 1.13%; APache KD anterograde: 6.82 ± 1.01%, *n* = 31–33 neurons, from *n* = 3 independent preparations). (**C**) Decreased average retrograde axonal velocity of mRFP-LC3b puncta in APache KD neurons compared to control neurons (control: 0.23 ± 0.02 μm/s; APache KD: 0.17 ± 0.02 μm/s, *n* = 25–28 puncta, from *n* = 3 independent preparations). (**D**) Time-lapse images of mRFP-TrkB positive puncta (arrowheads) along the axons of control or APache KD cortical neurons and representative kymographs on the right panels. Scale bar, 5 μm. (**E**) Relative axonal mobility of mRFP-TrkB puncta in control and APache KD neurons expressed in percent of total TrkB puncta (control static: 55.00 ± 4.95%; APache KD static: 83.95 ± 3.00%; control retrograde: 33.60 ± 4.67%; APache KD retrograde: 10.75 ± 2.07%; control anterograde: 8.75 ± 1.93%; APache KD anterograde: 5.25 ± 1.52%, *n* = 20 neurons, from *n* = 3 independent preparations). (**F**) Decreased average retrograde axonal velocity of mRFP-TrkB puncta in APache KD neurons compared to control neurons (control: 0.32 ± 0.02 μm/s; APache KD: 0.22 ± 0.02 μm/s, *n* = 50–58 puncta, from *n* = 3 independent preparations). All graphs show means ± SEM. ****p < 0.0001, two-way ANOVA/Bonferroni’s tests (**B**, **E**); *p < 0.05; ****p < 0.0001, Mann-Whitney’s *U*-test (**C**, **F**)

Efficient autophagosomal cargo degradation is dependent on functional retrograde transport to the cell body [32, 33]. To test whether a defective retrograde axonal transport underlies the impairment of autophagosome-lysosome fusion and causes autophagosome accumulation at the cell periphery in APache-silenced neurons, we monitored the axonal mobility of autophagosomes in control and APache KD neurons expressing RFP-LC3 by live-cell imaging, and quantitatively analyzed motility by generating kymographs (Fig. 7A). In control neurons, most of RFP-LC3-positive puncta were stationary, while motile vesicles moved predominantly in the retrograde direction and rarely in the anterograde direction (Fig. 7B). APache deletion significantly increased the stationary LC3 puncta fraction and concomitantly decreased the retrogradely moving fraction, leaving the anterograde autophagosome transport unaffected (Fig. 7B). Moreover, the loss of APache significantly reduced the velocity of the retrograde axonal transport of RFP-LC3-positive puncta compared to controls (Fig. 7C). To identify whether, akin to AP-2, APache also mediates retrograde transport of TrkB-containing autophagosomes, we performed live-cell imaging to track the dynamics of mRFP-TrkB-positive puncta in control and APache KD neurons (Fig. 7D). APache loss significantly reduced the motility of mRFP-TrkB puncta, resulting in largely stationary vesicles at the expense of a decrease in retrograde mobility and average speed of retrograde transport (Fig. 7E, F). The data demonstrate that APache regulates retrograde trafficking of autophagosomes from distal axons to the cell soma. In addition, they support a functional cooperation between APache and AP-2 in controlling retrograde movement of TrkB-containing signaling amphisomes.

**Fig. 8:**
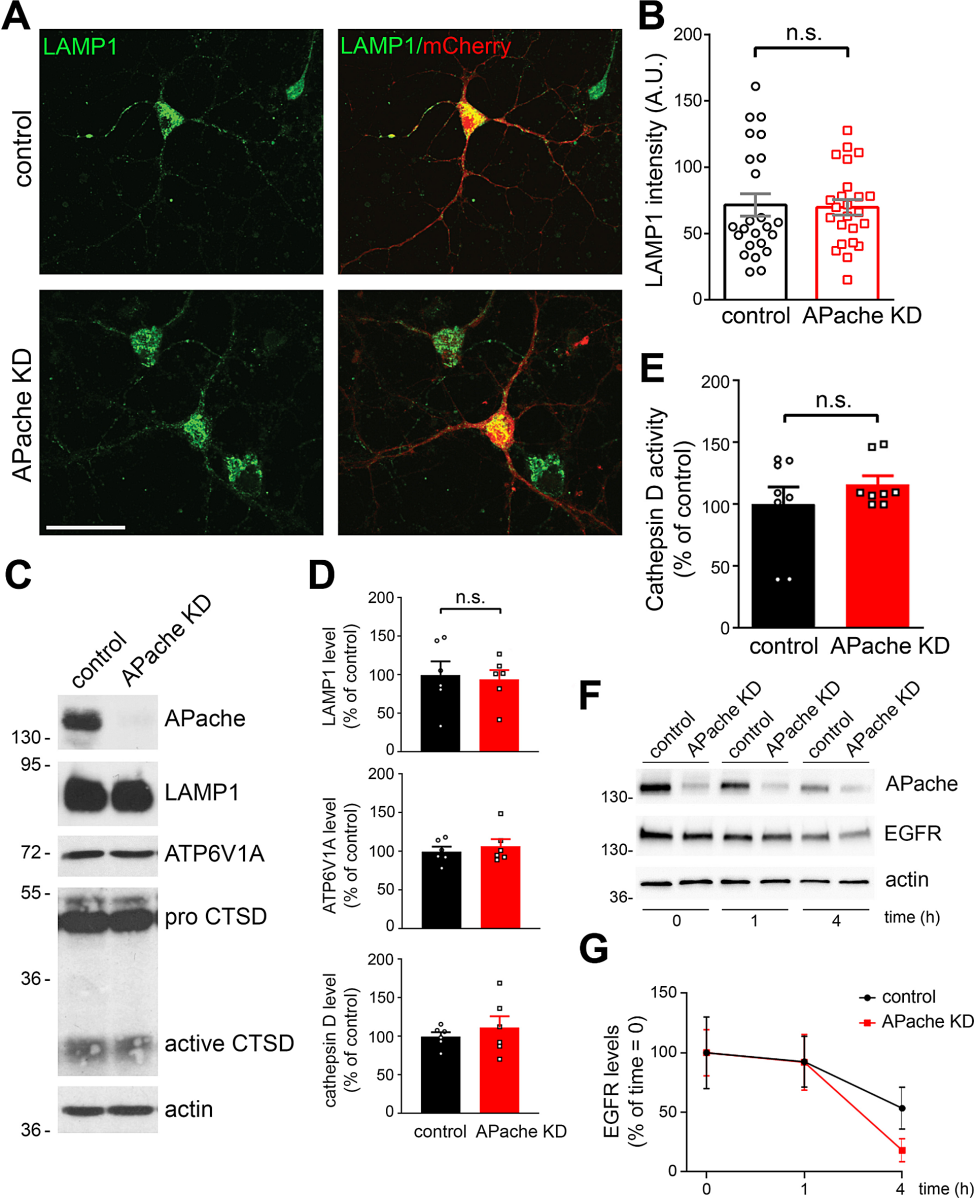
Lysosome density and function are not affected by APache silencing. (**A**) Representative confocal images of either control or APache KD neurons (mCherry-shRNA) stained for the lysosomal marker LAMP1 (green) at 17 DIV. Scale bar, 20 μm. (**B**) LAMP1 fluorescence intensity in APache KD neurons are unaltered compared to control neurons (control: 71.46 ± 8.44, APache KD: 69.70 ± 5.87, *n* = 24 neurons, from 3 independent preparations). A.U. = arbitrary units of fluorescence intensity. (**C**,** D**) Representative blots (**C**) and quantitative analysis (**D**) of LAMP1, ATP6V1A and cathepsin D (CTSD, pro-form + active form) protein levels in control and APache KD neurons at 17 DIV. Actin was used as loading control. No significant changes in protein expression levels were observed. Protein levels in APache KD neurons are expressed in percent of control neurons. (LAMP1: 97.71 ± 4.82%; ATP6V1A: 106.8 ± 9.14%; cathepsin D: 111.4 ± 14.82%, from *n* = 6 independent preparations). (**E**) Cathepsin D activity in control and APache KD neurons. Data are normalized to µg protein/sample and expressed in percent of control values (APache KD: 116.3 ± 6.97%, *n* = 8 samples from 3 independent preparations). (**F**) Representative blots of the EGFR degradation assay. Control and APache KD cortical neurons at 17 DIV were treated with EGF (200 ng/ml, 15 min) and incubated for the indicated times in the presence of CHX (5 µg/ml). Cell lysates were analyzed with anti-EGFR antibodies to monitor degradation of EGFR. Actin was used as loading control. (**G**) Quantitative analysis of EGFR protein levels, expressed in percent of the initial amount, revealed no significant changes in its degradation rate between control and APache KD neurons. (control 1 h: 92.49 ± 21.32%; APache KD 1 h: 92.12 ± 23.46%; control 4 h: 53.46 ± 17.52%; APache KD 4 h: 18.12 ± 9.72%, from *n* = 5 independent preparations). All graphs show means ± SEM. n.s.= non-significant; unpaired Student’s *t*-test with Welch’s correction (**B**, **D**, **E**); two-way ANOVA/Bonferroni’s tests (**G**)

However, a blockade of downstream steps in autophagy, such as an impairment of lysosomal degradation, would also slows down axonal transport of autophagosomes and worsen their accumulation within axons [34–36]. To further clarify this aspect, we analyzed the density and functionality of lysosomes in the neuronal soma. The immunoreactivity of LAMP1 (Fig. 8A, B), as well as its protein levels (Fig. 8C, D) were unaltered in APache KD neurons compared to control neurons. Moreover, the protein levels of ATP6V1A, the catalytic subunit A of the v-ATPase, the protein complex that acidifies lysosomes, as well as the levels and activity of the lysosomal cathepsin D hydrolase (CTSD) (Fig. 8C-E) were not significantly altered in APache-silenced neurons. To further analyze lysosomal function, we evaluated the EGF-induced degradation of EGF receptor. Again, APache KD cells revealed similar lysosomal degradative capacity compared to control neurons (Fig. 8F, G). Taken together, these results strongly support that the autophagosome accumulation occurring upon APache silencing is due to a blockade of autophagic flux caused by an impaired and slowed down retrograde transport of autophagosomes to the cell body, and not by impaired lysosomal function.

### APache is downregulated in sporadic AD brains

Since dysfunctional autophagy can hinder neuronal survival and be accompanied by cellular apoptosis, we analyzed the levels of the apoptotic marker active caspase-3 in total cell lysates by Western blot, and observed significantly increased protein levels in APache-depleted neurons compared to controls (Fig. S5 A, B). A link between neurodegenerative disorders and defective autophagosome transport [37] or p62/SQSTM1 accumulation [33] as a major pathological feature has been described, as well as a signal crosstalk between autophagy and apoptosis in regulating the pathogenesis of Alzheimer’s disease (AD) [38]. Given our evidence that APache depletion interferes with autophagosome trafficking and autophagic clearance of p62/SQSTM1 and initiates apoptosis signaling, we measured the expression of APache by immunoblotting in autopsy sections of the frontal cerebral cortex from a subset of late-onset sporadic AD patients and age-matched cognitively normally aging elderly subjects as controls [39]. Interestingly, although preliminary, we found that both APache and AP-2 levels were severely reduced in late-onset sporadic AD patients, while presynaptic markers such as the SV-associated proteins synaptophysin and synaptotagmin1 were still normal (Fig. S5 C, D).

## Discussion

Neuronal autophagy is essential for maintaining cellular homeostasis, function, and survival. Although the yeast core components of the autophagic machinery are conserved in mammals, additional regulators mediating and controlling autophagosome behavior have evolved in neurons, where the complex morphology and extended lifetime of the cells make the process highly specialized [7]. Understanding the processes and regulation of autophagy is important for understanding neurodevelopmental and neurodegenerative pathologies associated with autophagy dysfunctions [40–43]. Our study identifies APache, a neuron-specific protein involved in SV trafficking, as a regulator of the neuronal autophagy pathway, essential for retrograde transport of autophagosomes. Interestingly, APache is responsive to induction of autophagy by mTOR inhibition with Torin1 in primary cortical neurons.

In neurons, autophagosome formation and cargo degradation by fusion with lysosomes is a constitutive and spatially segregated process: autophagosomes form at synaptic terminals and mature, after fusion with late endosomes, becoming late-stage autophagosomes or amphisomes, that are retrogradely transported to the cell body where the majority of lysosomes are located [9, 44]. The molecular mechanisms governing autophagy and autophagosome maturation in neurons start to be clarified, and neuron-specific regulators, many of which are also involved in SV recycling, are being identified [11].

We have shown that in primary cortical neurons, APache depletion by RNAi blocks autophagic flux, as witnessed by the neuronal and synaptic accumulation of LC3-positive AVs, LC3- and Rab7-dually positive amphisomes, and p62/SQSTM1. Live imaging microscopy has also shown that APache, expressed in axonal processes and presynaptic terminals [25], plays a key role in coordinating the retrograde axonal transport of autophagosomes/TrkB-containing amphisomes toward the cell body, excluding its contribution in the early stages of autophagosome formation or in lysosomal clearance.

Several factors that function as scaffolding molecules have been implicated in the transport process that links autophagic and endocytic vesicles: the Rab7-interacting lysosomal protein RILP [26], the JIP family [45, 46], Snapin [47], AP-2 [21] and SIPA1L2 [48] can also play a role in autophagy by acting as distinct adaptor proteins or cooperating to coordinate the recruitment of the dynein/dynactin motor complexes. Most of these adaptors have also been described for the retrograde transport of TrkB, although the non-degradative function of autophagosomes in neuronal signaling is poorly described and many molecular aspects still need to be elucidated.

The precise molecular mechanism that mediates APache function, independent or not of its role in SV recycling, remains to be determined and future studies will be needed to explore it in detail. It is tempting to speculate that APache cooperates with AP-2 in regulating the retrograde transport of autophagosome/amphisomes, as several lines of experimental evidence in previous [25] and present works point to a close structural and functional interaction between the two proteins. Firstly, APache colocalizes with AP-2 in cortical neurons and co-immunoprecipitates from the mouse brain with AP-2α and β subunits and with p150^Glued^ subunits of dynactin (a protein we previously identified as specific APache-binding partner by mass spectrometry analysis), suggesting that APache participates in the macromolecular complex of interactions regulating autophagosome trafficking. AP-2 forms a protein complex with LC3 and dynactin/p150^Glued^ [21] and APache is also enriched in the vacuolar fraction positive for LC3II and LAMP1. Secondly, APache is associated and co-traffics with LC3 on autophagosomes in a predominant retrograde direction. Thirdly, the AP-2 expression levels are significantly reduced in both developing and mature APache-silenced neurons, over time windows in which autophagy plays essential roles for cellular growth, function, and viability, and the overexpression of the adaptor protein can revert the blockade of the autophagic flux induced by APache depletion. Finally, not only APache expression, but also AP-2 expression is upregulated after autophagy induction. Stimulation of autophagy by mTOR inhibition results in increased LC3II, AP-2 and APache levels, also at synaptic terminals, associated with an enhanced APache recruitment to LC3-positive AVs. The increased colocalization with LC3 suggests that APache and AP-2 may be upregulated at synapses to participate in the transport of autophagosomes toward the cell soma, as recently showed for the dynein adaptor RILP, whose expression and behavior are also controlled by mTOR [26].

In the absence of APache, autophagosomes turnover is dramatically reduced and a massive accumulation of autophagosomes/TrkB-containing signaling amphisomes occurs at synaptic boutons and in axons, a phenotype very similar to that observed in AP-2 KO neurons [21]. Autophagosome accumulation and defective cargo degradation result from inefficient initiation of retrograde transport of autophagosomes from distal axons, consistent with the decreased retrograde autophagosome motility observed in APache-depleted neurons. While we can exclude the contribution of lysosomal defects to the impaired maturation of autophagic vacuoles, a potential role of APache in the fusion of mature autophagosomes with lysosomes cannot be ruled out, considering the presence of clathrin coats assemblies on lysosomes [49] and the function of AP-2 in the SNARE complex formation.

Can a dysfunction in the APache gene be involved in the pathogenesis of neurodegenerative disorders associated with dysfunctional autophagy such as AD? While there are no reports of loss-of-function mutations of the APache gene in AD patients, its transcription was significantly reduced in frontotemporal lobar degeneration associated with the *MAPT* p.R406W mutation and supranuclear palsy [50]. Moreover, also AP-2 was found decreased in human iPSC-derived neurons from patients with late-onset AD [51]. Here, we describe that APache depletion impairs autophagy and causes neuronal apoptosis, likely through an activation of the transcriptional repressor BCLAF1 (Bcl-2 associated transcription factor 1) that is a potential APache interactor [25]. Interestingly, we found that the expression of both APache and AP-2 proteins were significantly decreased in the frontal cerebral cortex of a subset of late-onset sporadic AD patients with respect to cognitively normally aging elderly subjects. Although preliminary, these data lead to the possibility that altered expression or function of APache and the ensuing loss of interaction with AP-2 might be one of the underlying causes of the impaired autophagy and early synaptic alterations observed in AD [52–54].

Although further studies are needed to unravel whether APache exerts a stabilizing role on the formation of the AP-2/LC3/dynactin complex or additional distinct functions, our results strongly support a role for APache in the regulation of autophagosome retrograde transport to maintain neuronal homeostasis and foster further research addressing the physiological, pathological, and therapeutic implications of these findings.

## Materials and methods

### Constructs

APache protein is encoded by the *KIAA1107* gene. For detailed description of constructs used for APache silencing see Piccini et al. (2017) [25]. Briefly, shRNA targeting the 3’ untranslated region (UTR) of the mouse *Kiaa1107* transcript and control shRNA (Luciferase shRNA; Sigma-Aldrich) were inserted into the pLKO.1-CMV-mCherry lentiviral vectors. For rescue experiments, the p277-EGFP-APache vector was used, being intrinsically resistant to APache-shRNA. For APache silencing through transfection, APache shRNA and control shRNA were inserted into pLKO.1-CMV-eGFP or pLKO.1-CMV-mTourquoise vectors. Expression plasmid encoding EGFP-APache was described previously (Piccini et al., 2017). mRFP-TrkB was a kind gift from Dr. Natalia Kononenko (CECAD, University of Cologne, Germany). AP-2µ2-mCherry was a gift from Christien Merrifield (Addgene plasmid # 27672; http://n2t.net/addgene:27672; RRID: Addgene 27672), pBABE-puro mCherry-EGFP-LC3B was a gift from Jayanta Debnath (Addgene plasmid # 22418; http://n2t.net/addgene:22418; RRID: Addgene_22418) and pTagRFP-C-LC3b (#FP141) was from Evrogen.

### Transfection and transduction of primary neurons

Cultures of dissociated primary cortical neurons were prepared from embryonic 17/18-day (E17–18) brains of C57BL/6J mice (Charles River, Calco, Italy) and plated on poly-L-lysine (0.1 mg/ml, #25988-63-0, Sigma-Aldrich)-coated 25-mm glass coverslips at low density (6 × 10^4^ cells/coverslip) and on poly-L-lysine (1 mg/ml)-coated 35-mm wells at high density (0.3–1 × 10^6^ cells/well). Cells were maintained in a culture medium consisting of Neurobasal (#21103-049, Gibco) supplemented with B-27 (1:50 v/v, Gibco #17504-007, Gibco), Glutamax (1% w/v, #35050-38, Gibco), penicillin–streptomycin (1%, #10378-016, Gibco) and kept at 37 °C in a 5% CO_2_ humidified atmosphere. Under this culture condition approximately 85–90% of the cortical neurons are glutamatergic and cultures are almost glia-free [55]. All experiments were carried out in accordance with the guidelines established by the European Community Council (Directive 2010/63/EU of March 4, 2014) and were approved by the Italian Ministry of Health (authorization n. 600/2020-PR). For lentiviral infection experiments, neurons were transduced with 5 multiplicity of infection (MOI) of lentiviral vectors into cell medium at 12 days in vitro (DIV). After 24 h, half of the medium was replaced with fresh medium, and experiments were performed 5 days post infection. For rescue experiments, neurons were co-transduced with 5 + 5 MOI of lentiviral vectors. For plasmid and shRNA transfection experiments, neurons were transfected at 11–14 DIV using Lipofectamine™ 2000 (#11668019, Thermo Fisher Scientific) according to the manufacturer’s instructions and analyzed 3 days later. When specified, neurons were treated for 4 h at 37 °C with 250 nM Torin1 (#2273, BioVision) and/or 5 µg/ml cycloheximide (#C7698, Sigma-Aldrich), or an equivalent volume of DMSO/Milli-Q water as a solvent control.

### Immunocytochemistry and fluorescence microscopy

Low-density primary cortical neurons were fixed at 17 DIV in 4% paraformaldehyde/4% sucrose in phosphate-buffered saline (PBS), pH 7.4 for 15 min at room temperature, permeabilized with 0.1% Triton X-100 in PBS for 5 min and blocked with 0.1% Triton X-100, 3% fetal bovine serum in PBS for 30 min. Samples were incubated with the following primary antibodies diluted in blocking solution for 2 h at room temperature or overnight at 4 °C: rabbit anti-APache (1:500, PRIMM EFA/10 201010-00019), rabbit anti-LAMP1 (1:200, #L1418, Sigma-Aldrich), rabbit anti-LC3B (1:200, #L7543, Sigma-Aldrich), mouse anti-LC3B (1:100, #AM20213PU-N, Origene), rabbit anti-p62/SQSTM1 (1:500, #P0067, Sigma-Aldrich), mouse anti-pan-axonal neurofilament marker (1:500, #SMI-312R, Covance), rabbit anti-Rab7 (1:250, #ab137029, Abcam), mouse anti-VAMP2 (1:500, #104211, Synaptic Systems), guinea pig anti-VAMP2 (1:500, #1042, Synaptic Systems). Immunostaining was detected using Alexa 488, and/or 647-Fluor-conjugated secondary antibodies (1:500, Thermo Fisher Scientific) diluted in blocking solution for 1 h at room temperature. Samples were mounted in Prolong Gold Antifade reagent with or without 4′,6′-diamidino-2- phenylindole (DAPI) (Thermo Fisher Scientific, respectively #P36934 and #P36935). Confocal images were acquired with a confocal laser-scanning microscope (SP8, Leica Microsystems GmbH, Wetzlar, Germany) with a 40X/1.4 oil-immersion objective. Each image consisted of a stack of images taken through the z-plane of the cell acquired every 300 nm. For each set of experiments, all images were acquired using the same optical acquisition settings for all conditions, the offset parameters in same cases were adjusted to better visualize and isolate APache and LC3 synaptic staining. Offline analysis was performed using ImageJ/FiJI and mean fluorescence intensities were quantified on ROIs manually selected on the cell soma or automatically detected using the “Analyze Particle” tool of ImageJ for synaptic boutons detection. VAMP2-positive puncta with areas of 0.1–2 µm^2^ were considered *bona fide* synaptic boutons. Co-localization studies were performed using the “JACoP” co-localization plug-in and calculating the Manders’ coefficient (shown in percent on the graph) in ROIs manually selected on the cell soma or at VAMP2-positive puncta using the ImageJ selection tool. For the analysis of LC3- or Rab7-positive puncta along neurites, a constant threshold was applied to all images and the number of fluorescent puncta was determined using the “Analyze particles” tool of ImageJ in manually selected ROIs (30-µm length). For the analysis of the mCherry-eGFP-LC3B tandem assay, the ratio of the mean fluorescence intensity of eGFP over mCherry was calculated at the cell soma. For quantitative analysis of the density of VAMP2-positive puncta, masks of mCherry-infected neurons were created using ImageJ/FiJI and the VAMP2 signal was thresholded using the same parameters for all the images. Colocalization points between the mCherry mask and VAMP2 signal, identified with the ImageJ/FIJI “JACoP” co-localization plug-in, represent VAMP2-positive *bona fine* synaptic puncta of infected neurons. The density of VAMP2-positive puncta was calculated as the number of puncta normalized on the mCherry neuronal area.

### Live-cell neuron imaging and analysis

Low-density primary cortical neurons co-transfected with EGFP-APache and mRFP-LC3 or either control eGFP-shRNA (control) or eEGFP-APache-shRNA (APache KD) and mRFP-LC3b or mRFP-TrkB were imaged 3 days later in Tyrode’s solution (10 mM glucose, 140 mM NaCl, 2.4 mM KCl, 10 mM Hepes, 2 mM CaCl_2_, 1 mM MgCl_2_) in an environmental chamber at temperature-controlled stage (37 °C) on an inverted epifluorescence microscope (Olympus IX81, Olympus Corporation, Tokyo, Japan) with a 60X oil-immersion objective. Axons were identified based on morphological parameters [56]. Time-lapse recordings were acquired at 0.25–1 Hz for 30–120 s with Olympus xCellence rt Software. Frame rates were optimized for each experimental approach to capture events with high time resolution while minimizing photobleaching. Within each experiment, all conditions were imaged with the same paradigm. Motility of mRPF-LC3 or mRFP-TrkB puncta was analyzed by generating kymographs using “KymographBuilder” plug-in in ImageJ/FIJI. Vesicles were manually tracked with the “Manual Tracking” plug-in in ImageJ/FIJI. Retrograde and anterograde motile mRFP-LC3b or mRFP-TrkB puncta were identified by the respective location of the cell soma or axonal tip during imaging. The retrograde mean speed of individual fluorescent puncta was calculated by determining the net displacement over the time-lapse. Stationary puncta were defined as those moving at a velocity < 0.05 μm/s over the imaging period.

### Transmission electron microscopy (TEM)

Low-density cultures of cortical neurons were infected at 12 DIV with either control shRNA or APache shRNAs plus APache-EGFP in rescue experiments and processed for TEM. Neurons were fixed at 17 DIV with 1.2% glutaraldehyde in 66 mM sodium cacodylate buffer, post-fixed in 1% OsO_4_, 1.5% K_4_Fe(CN)_6_, 0.1 M sodium cacodylate, *en bloc* stained with 10% of uranyl acetate replacement stain (EMS) for 30 min, dehydrated, and flat embedded in epoxy resin (Epon 812, TAAB). After baking for 48 h, the glass coverslip was removed from the Epon block by thermal shock and neurons were identified by means of a stereomicroscope. Embedded neurons were then excised from the block and mounted on a cured Epon block for sectioning using an EM UC6 ultramicrotome (Leica Microsystems). Ultrathin Sects. (60–70 nm thick) were collected on 200-mesh copper grids (EMS) and observed with a JEM-1011 electron microscope (Jeol, Tokyo, Japan) operating at 100 kV using an ORIUS SC1000 CCD camera (Gatan, Pleasanton, CA). For each experimental condition, at least 50 images of synapses and neurites were acquired at 10,000x magnification (sampled area per experimental condition: 36 µm^2^). Synaptic profile area, AV number and density were determined using ImageJ. AVs were defined as single or double membrane-bound vacuoles containing intracellular material.

### Western blot analysis

Total cell lysates from high-density cortical neuronal cultures at 17 DIV were extracted in lysis buffer (150 mM NaCl, 50 mM Tris-HCl pH 7.4, 1 mM EDTA pH 8, 1% Triton X-100) supplemented with protease and phosphatase inhibitors cocktail (respectively #58715 and #58705, Cell Signaling). After 10 min of incubation on ice, lysates were collected and clarified by centrifugation (10 min at 1,000 x g at 4 °C). For digitonin-based membrane/organelle-enriched and cytosol fractionation, neurons were washed in HBSS and permeabilized in buffer D (modified from 57): 0.02% Digitonin (#D-5628, Sigma-Aldrich), 300 mM sucrose, 5 mM Hepes, 100 mM NaCl, 5 mM EDTA, 3 mM MgCl_2_ supplemented with protease inhibitor cocktail under gentle shaking at 4 °C for 20 min. Supernatant (cytosolic fraction) was collected, and membranes were scraped from the well plate in buffer D. Protein concentration was determined using either Bradford Protein Assay (#5000006, Bio-Rad) or Pierce™ BCA Protein Assay Kits (#23227, Thermo Fisher Scientific) assay, and equivalent amounts of protein were subjected to SDS-PAGE on polyacrylamide gels (8-14%) and blotted onto nitrocellulose membranes (Whatman). Blotted membranes were blocked for 1 h in 5% milk in Tris-buffered saline (10 mM Tris, 150 mM NaCl, pH 8.0) plus 0.1% Triton X-100 and incubated 2 h at room temperature or overnight at 4 °C with the following primary antibodies: mouse anti-actin (1:1000, #A4700, Sigma-Aldrich), rabbit anti-APache (1:1000, PRIMM EFA/10 201010-00019), mouse anti-AP-2α (1:1000, #610502, BD Transduction Laboratories), rabbit anti-AP2β (1:2000, #NBP2-15563, Novus Biologicals), mouse anti-AP2µ (1:1000, #611350, BD Transduction Laboratories), rabbit anti-ATG3 (1:1000, #3415P, Cell Signaling), rabbit anti-ATG5 (1:1000, #8540P, Cell Signaling), rabbit anti-ATP6V1A (1:1000, #ab137574, Abcam), rabbit anti-caspase-3 (1:1000; #9662, Cell Signaling), rabbit anti-cathepsin D (1:2000, #219361, Millipore), rabbit anti-EGFR (1:1000, #ab52894, Abcam), rabbit anti-GAPDH (1:5000, #2118, Cell Signaling), rabbit anti-LAMP1 (1:1000, #ab24170, Abcam), rabbit anti-LC3B (1:1000, #L7543, Sigma-Aldrich), rabbit anti-mTOR (1:1000, #2983, Cell Signaling), rabbit anti-phospho-mTOR Ser2448 (1:1000, #5536, Cell Signaling), mouse anti-NaK3 (ATP1A3) (1:1000, #MA3-915, Invitrogen), mouse anti p150/Glue (1:1000, #610474, BD Transduction Laboratories), rabbit anti-Rab5 (1:1000, #ab18211, Abcam), mouse anti-Rab7 (1:1000, #ab50533, Abcam), mouse anti-synaptophysin (anti-SYP; 1:5000, #101011, Synaptic Systems), mouse anti-synaptotagmin1 (anti-Syt1; 1:1000, #105011, Synaptic System), rabbit anti-ULK1 (1:1000, #8054, Cell Signaling), rabbit anti-phospho-ULK1 Ser757 (1:1000, #6888, Cell Signaling), mouse anti-vinculin (1:500, #V9264, Sigma-Aldrich). Membranes were washed and incubated for 1 h at room temperature with peroxidase-conjugated goat anti-mouse ((#1706516, 1:3000; Bio-Rad) or anti-rabbit (#1706515, 1:5000; Bio-Rad) secondary antibodies. Immunoreactivity was detected using the ECL chemiluminescent detection system (#32106, Thermo Fisher Scientific) and autoradiography or by ChemiDoc Imaging System (Bio-Rad Laboratories, Hercules, CA, USA). Quantification was performed by bands densitometric analysis with ImageJ/FIJI for Windows (Rasband, W.S., ImageJ, U. S. National Institutes of Health, Bethesda, MD, USA). For quantification, protein levels were always first normalized to the loading control from each corresponding lane and then the levels in the treated/APache KD neurons were normalized relative to controls set to 100%.

### Immunoprecipitation assay

Mouse brain was homogenized in ice-cold buffered sucrose solution (0.32 M sucrose, 5 mM HEPES, pH 7.4) plus 100 mM NaCl and protease inhibitors and cleared by low-speed centrifugation (1,000 × g for 10 min at 4 °C). The supernatant was incubated with 1% Triton X-100 for 30 min at 4 °C and then centrifuged at 150,000 x g for 45 min at 4 °C. Equivalent amounts of brain extract were incubated for 3 h at 4 °C with either rabbit anti-APache or rabbit control IgG (10 µg/sample) pre-coated overnight with Protein G-Sepharose (GE Healthcare). After extensive washes, samples were resolved by SDS–PAGE and analyzed by Western blotting.

### Cathepsin D activity assay

Cellular Cathepsin D (CTSD) activity was determined using a CTSD activity assay kit (#K143, BioVision), according to manufacturer’s protocol. The assay is based on the use of the fluorescently labeled preferred CTSD substrate sequence GKPILFFRLK(Dnp)-DR-NH_2_ that emits fluorescence after cleavage. Briefly, transduced primary cortical neurons were lysed at 17 DIV in 200 µl of chilled CD Cell Lysis Buffer, incubated on ice for 10 min, and centrifuged for 5 min at top speed. Ten µg /well of cell lysate were mixed with 50 µl of Reaction Buffer and 2 µl of substrate to a final volume of 102 µl into a 96-well plate and incubated for 2 h at 37 °C. Samples were read in triplicate at 10-min intervals (Ex/Em = 328/393 nm) at multiplate TECAN^®^ reader (Tecan Trading AG, Switzerland) using a 320 ± 25-nm excitation filter and 485 ± 20-nm emission filter. Background values were calculated by reading wells filled only with solutions and subtracted to each sample value. Data were normalized to µg protein/sample and expressed in percentage of control value.

### Epidermal growth factor receptor degradation assay

Epidermal growth factor receptor (EGFR) degradation assay was performed as previously described [58] with minor modifications. High-density cortical neurons at 17 DIV were incubated with 200 ng/ml EGF (#PHG0313, Thermo Fisher Scientific) for 15 min at 4 °C allowing ligand-receptor binding. To synchronize ligand-receptor internalization, the ligand-containing medium was replaced by fresh prewarmed medium. Cells were incubated at 37 °C allowing ligand-receptor internalization for 0, 1 and 4 h in the presence of 5 µg/ml cycloheximide to inhibit the *de novo* synthesis of EGFR. Total cell lysates were analyzed by Western blot using an anti-EGFR antibody to monitor degradation of EGFR band.

### Human brain tissue

We used previously characterized [39] frozen blocks and formalin-fixed autopsy sections of the frontal cerebral cortex from 8 cases with late-onset sporadic Alzheimer’s disease (AD) (mean age at death 80 ± 8 years; post-mortem interval 8 h ± 3; clinical history of disease; pathological diagnosis according to the Consortium to Establish a Registry for Alzheimer’s Disease (CERAD) criteria) provided by the brain bank of Case Western Reserve University, Cleveland, OH, and from 8 cognitively normally aging elderly subjects as controls (mean age at death 83.3 ± 10 years, post-mortem interval 9.5 h ± 4). The latter subjects had been tested neuropsychologically annually and agreed to be autopsied for research purposes (provided by the Alzheimer’s Disease Research Center, University of Kentucky). Their neuropsychological scores were within the normal range; in the cerebral cortex abundant amyloid plaques were present with absent or scarce neurofibrillary pathology. Protein lysates from autopsy sections were extracted in ice-cold buffered sucrose solution (0.32 M sucrose, 5 mM Hepes, pH 7.4) supplemented with 100 mM NaCl and protease inhibitors cocktail and centrifuged at 1,000 x g for 10 min at 4 °C to obtain a post-nuclear supernatant fraction.

### Statistical analysis

Normal distribution of experimental data was assessed using the D’Agostino-Pearson’s normality test (*n* > 6) or Shapiro-Wilk test (*n* ≤ 6). To compare two normally distributed sample groups, the unpaired Student’s *t*-test with the Welch’s correction was used. To compare two sample groups that were not normally distributed, the nonparametric Mann–Whitney’s *U*-test was used. To compare more than two normally distributed sample groups, we used one- or two-way ANOVA followed by the Bonferroni’s multiple comparison test. In cases in which more than two sample groups were not normally distributed, we used the Kruskal–Wallis’s ANOVA test followed by the Dunn’s multiple comparison test. Statistical analysis was carried out using Prism 7.0 software (Graphpad Software Inc., La Jolla, CA). Significance level was preset to *p* < 0.05. Data are expressed as means ± standard error of the mean (SEM) for number of cells/samples or independent preparations (n) as detailed in the figure legends.

## Electronic supplementary material

Below is the link to the electronic supplementary material.

Supplementary Material 1

## Data Availability

The datasets generated and analyzed during the current study are available from the corresponding author on reasonable request.
