# Correction: Apache is a neuronal player in autophagy required for retrograde axonal transport of autophagosomes

**DOI:** 10.1007/s00018-025-05675-z

**Published:** 2025-07-30

**Authors:** Barbara Parisi, Alessandro Esposito, Enrico Castroflorio, Mattia Bramini, Sara Pepe, Antonella Marte, Fabrizia C. Guarnieri, Flavia Valtorta, Pietro Baldelli, Fabio Benfenati, Anna Fassio, Silvia Giovedì

**Affiliations:** 1 Dipartimento di Medicina Sperimentale, Università degli Studi di Genova, Genova, Italia; 2 Center for Synaptic Neuroscience and Technology, Italian Institute of Technology, Genoa, Italy; 3 IRCCS, Ospedale Policlinico San Martino, Genova, Italia; 4 Division of Neuroscience, IRCCS San Raffaele Scientific Institute, Milan, Italy; 5 Vita-Salute San Raffaele University, Milan, Italy; 6 Present Address: IRCSS, Ospedale Policlinico San Martino, Genova, Italy; 7 Present Address: Institute of Neuroscience, National Research Council (CNR), Vedano al Lambro, Italy; 8 Present Address: Department of Cell Biology, Universidad de Granada, Granada, Spain; 9 Department of Experimental Medicine, University of Genoa, Viale Benedetto XV, 3, 16122 Genova, Italy

**Correction: Cellular and Molecular Life Sciences (2024) 81:416** 10.1007/s00018-024-05441-7

In this article the affiliation details for Author Barbara Parisi were incorrectly given as

Dipartimento di Medicina Sperimentale, Università degli Studi di Genova, Genova, Italia and

Present Affiliation: Department of Cell Biology, Universidad de Granada, Granada, Spain but should have been Dipartimento di Medicina Sperimentale, Università degli Studi di Genova, Genova, Italia and IRCSS, Ospedale Policlinico San Martino, Viale Benedetto XV, 3, Genova 16122, Italy

The affiliation details for Author Alessandro Esposito were incorrectly given as

Dipartimento di Medicina Sperimentale, Università degli Studi di Genova, Genova, Italia and

Present Affiliation: Department of Cell Biology, Universidad de Granada, Granada, Spain but should have been Dipartimento di Medicina Sperimentale, Università degli Studi di Genova, Genova, Italia and ‘Present Affiliation: IRCSS, Ospedale Policlinico San Martino, Genova, Italy’.

The affiliation details for Author Mattia Bramini were incorrectly given as Center for Synaptic Neuroscience and Technology, Italian Institute of Technology, Genoa, Italy and IRCSS, Ospedale Policlinico San Martino, Viale Benedetto XV, 3, Genova 16122, Italy but should have been

Center for Synaptic Neuroscience and Technology, Italian Institute of Technology, Genoa, Italy and

Present Affiliation: Department of Cell Biology, Universidad de Granada, Granada, Spain

The affiliation details for Author Fabrizia C. Guarnieri were incorrectly given as

Division of Neuroscience, IRCCS San Raffaele Scientific Institute, Milan, Italy,

Vita-Salute San Raffaele University, Milan, Italy and IRCSS, Ospedale Policlinico San Martino, Viale Benedetto XV, 3, Genova 16122, Italy but should have been

Division of Neuroscience, IRCCS San Raffaele Scientific Institute, Milan, Italy

Vita-Salute San Raffaele University, Milan, Italy and ‘Present Affiliation: IRCSS, Ospedale Policlinico San Martino, Genova, Italy’.

In this article the 7th affiliation were incorrectly given as

‘IRCSS, Ospedale Policlinico San Martino, Viale Benedetto XV, 3, Genova 16122, Italy’

but should have been

‘Present Affiliation: IRCSS, Ospedale Policlinico San Martino, Genova, Italy’.

The original article has been corrected.

