# Supplementary material for: Apache is a neuronal player in autophagy required for retrograde axonal transport of autophagosomes
Source: Cell Mol Life Sci. 2024 Oct 5;81(1):416. doi: 10.1007/s00018-024-05441-7 (PMC11455771; doi:10.1007/s00018-024-05441-7)
Supplement: Supplementary file 1 — Supplementary Material 1 [file 18_2024_5441_MOESM1_ESM.docx]

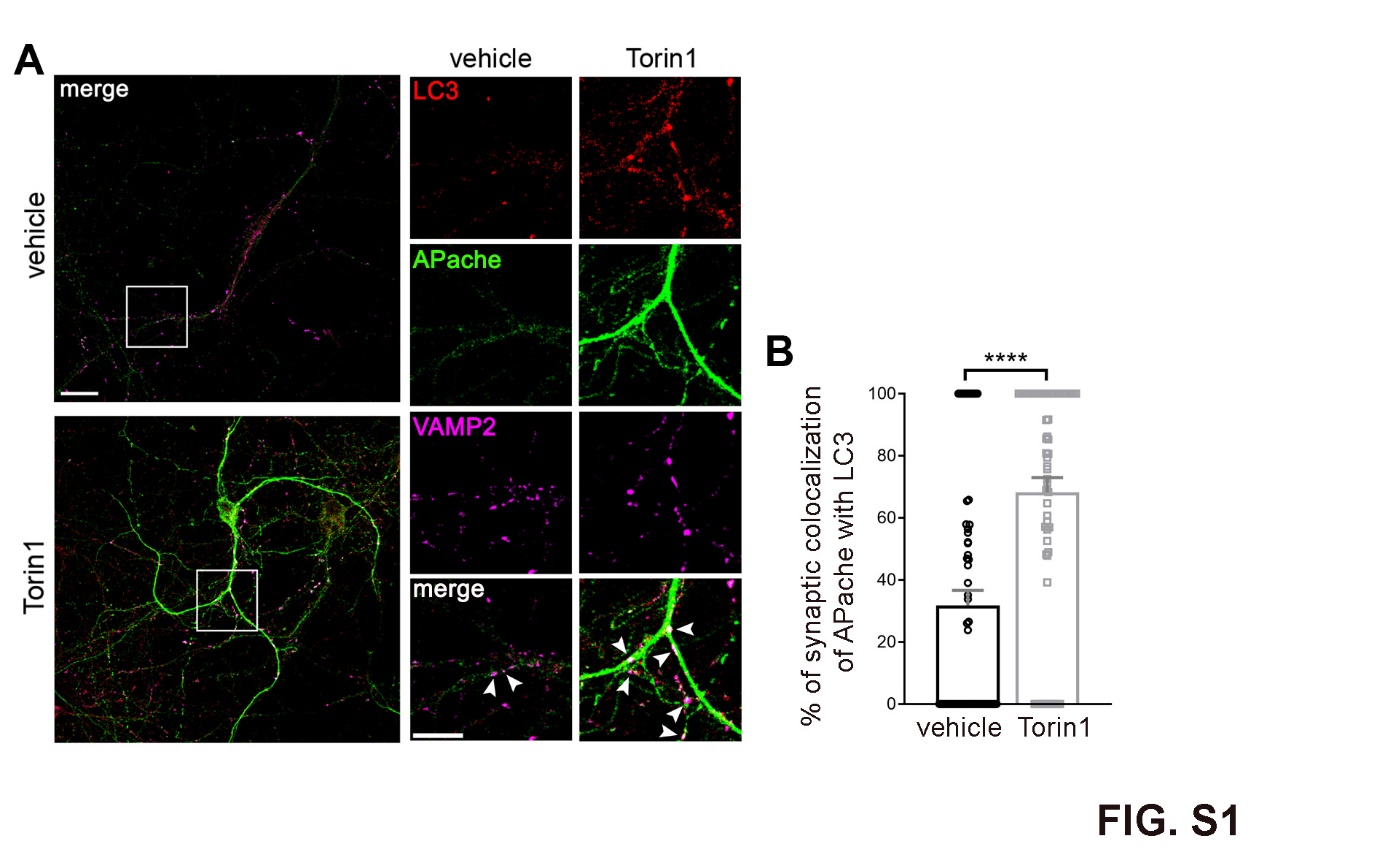


**Fig. S1 Increased synaptic colocalization of APache with LC3 after autophagy stimulation.**

**(A)** Representative confocal images of cortical neurons treated with either vehicle or Torin1 and triple stained for LC3 (red), APache (green) and VAMP2 (magenta) to identify synaptic boutons at 17 DIV. White boxes indicate panels magnified to the right. Arrowheads in the magnified inserts denote synaptic boutons positive for LC3 and APache. Scale bars: 20 µm, 10 µm (inserts). **(B)** Quantification of the percent of APache colocalizing with LC3 at VAMP2-positive puncta in vehicle- and Torin1-treated synapses based on Manders’ coefficient (vehicle: 31.76 ± 4.90%; Torin1: 68.26 ± 4.69%, n = 64–66 synapses, from 3 independent preparations). Graph shows means ± SEM. ****p < 0.0001, Mann-Whitney’s *U*-test.


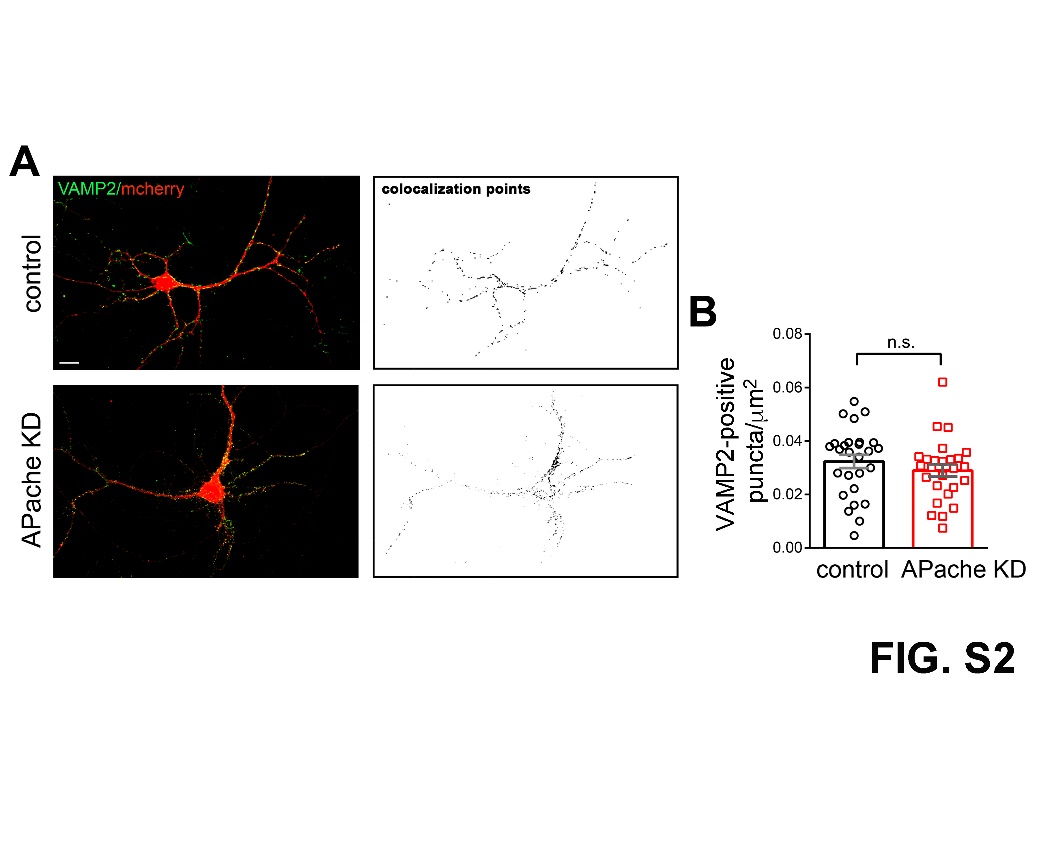


**Fig. S2 Unaltered** **density of synaptic boutons after APache silencing.**

**(A)** Representative images of control or APache KD cortical neurons (mCherry-shRNA) stained for VAMP2 (green) at 17 DIV. Co-localization panels indicate *bona fide* presynaptic boutons in transduced neurons. Scale bar, 10 µm. **(B)** Density of VAMP2-positive puncta in control and APache KD neurons (control: 0.032 ± 0.0024; APache KD: 0.029 ± 0.0023, n = 26–27 neurons, from 3 independent preparations). Graph shows means ± SEM. n.s.= non-significant, Mann-Whitney’s *U*-test.


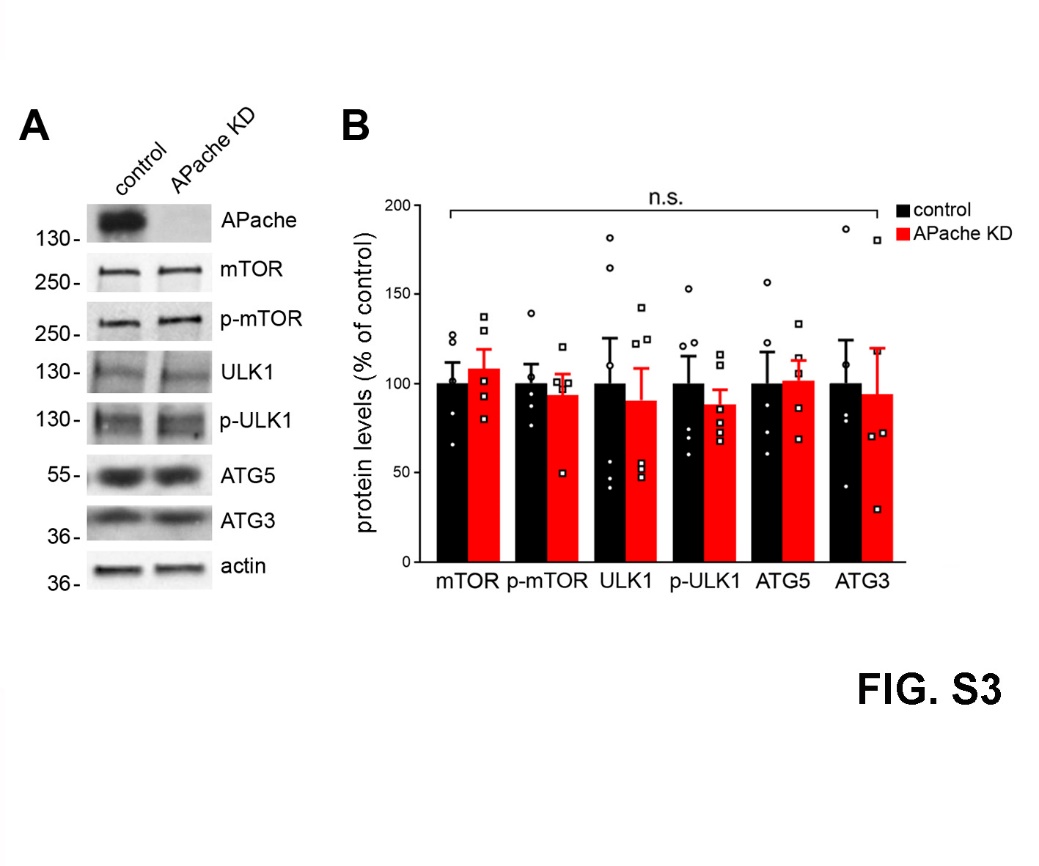


**Fig. S3 The impaired autophagic flux in the absence of APache does not result from decreased mTOR signalling.**

Representative blots **(A)** and quantitative analysis **(B)** of the expression levels of both total and active phospho-mTOR-Ser2448 (p-mTOR), total and active phospho-ULK1-Ser757 (p-ULK1), ATG5 and ATG3 in control and APache KD neurons at 17 DIV. No significant changes in protein expression levels were observed. Actin was used as loading control. Protein levels in APache KD neurons are expressed in percent of control neurons. (mTOR: 108.08 ± 10.93%; p-mTOR: 93.45 ± 11.72%; ULK1: 90.56 ± 17.75%; p-ULK1: 88.23 ± 8.24%; ATG5: 101.6 ± 11.18%; ATG3: 93.98 ± 25.71%, from n = 5–6 independent preparations). Graph shows means ± SEM. n.s.= non-significant, Unpaired Student’s *t*-test with Welch’s correction/Mann-Whitney’s *U*-test.


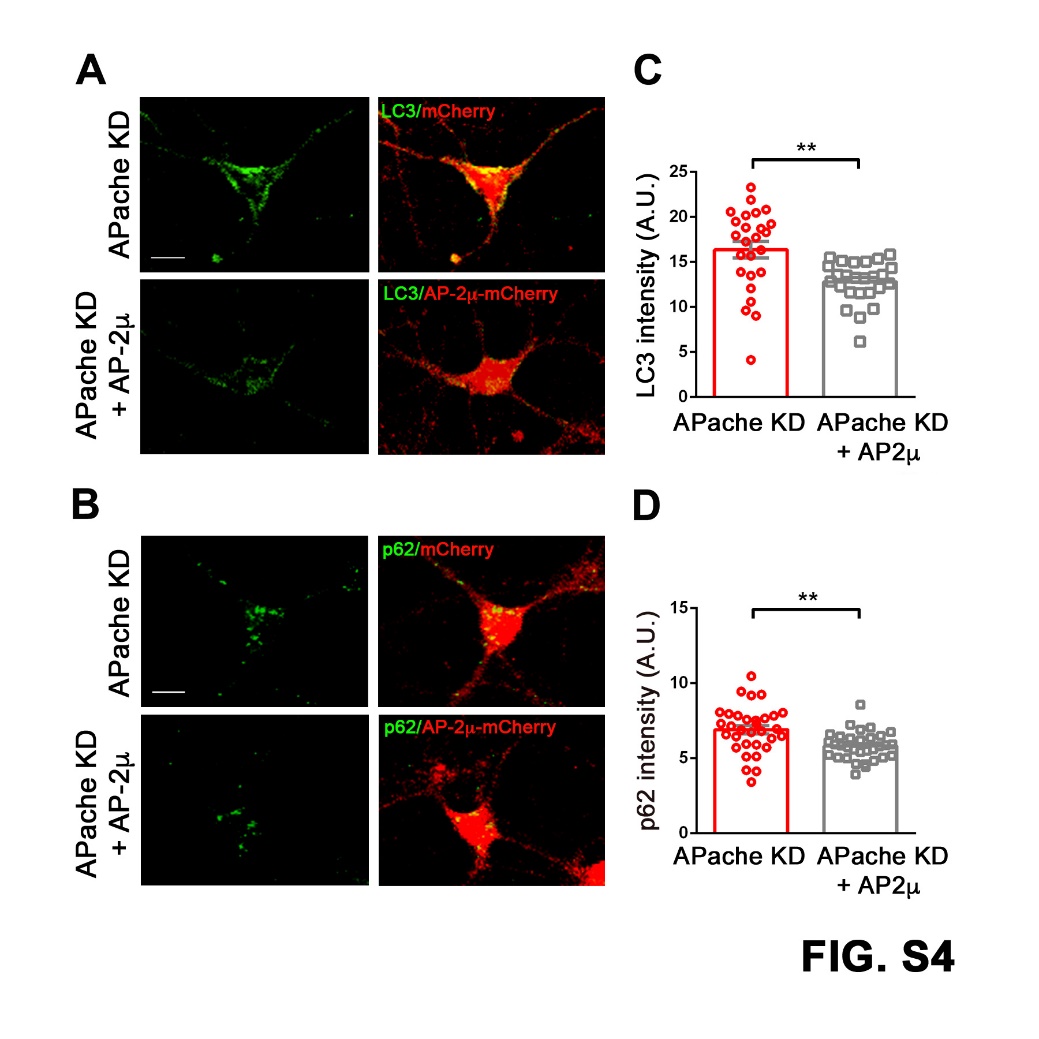


**Fig. S4. Overexpression of AP-2μ decreases the level of LC3 and p62/SQSTM1 in APache-silenced neurons.**

**(A,B)** Representative confocal images of mouse cortical neurons co-transfected with vectors coding for APache mTourquoise-shRNA (APache KD) and either AP-2μ-mCherry or empty vector at 14 DIV and stained for LC3 (green, **A**) or p62 (green, **B**) at 17 DIV. Scale bar, 10 µm. **(C,D)** Quantification of LC3 **(C)** and p62 **(D)** fluorescence intensity in APache KD neurons and after AP-2μ overexpression (LC3 APache KD: 16.37 ± 0.92; LC3 APache KD + AP-2μ: 12.81 ± 0.46; p62 APache KD: 6.91 ± 0.28; p62 APache KD + AP-2μ: 5.82 ± 0.17, n = 25–32 neurons, from 3 independent preparations). A.U. = arbitrary units of fluorescence intensity. All graphs show means ± SEM. **p < 0.01, unpaired Student’s *t*-test.


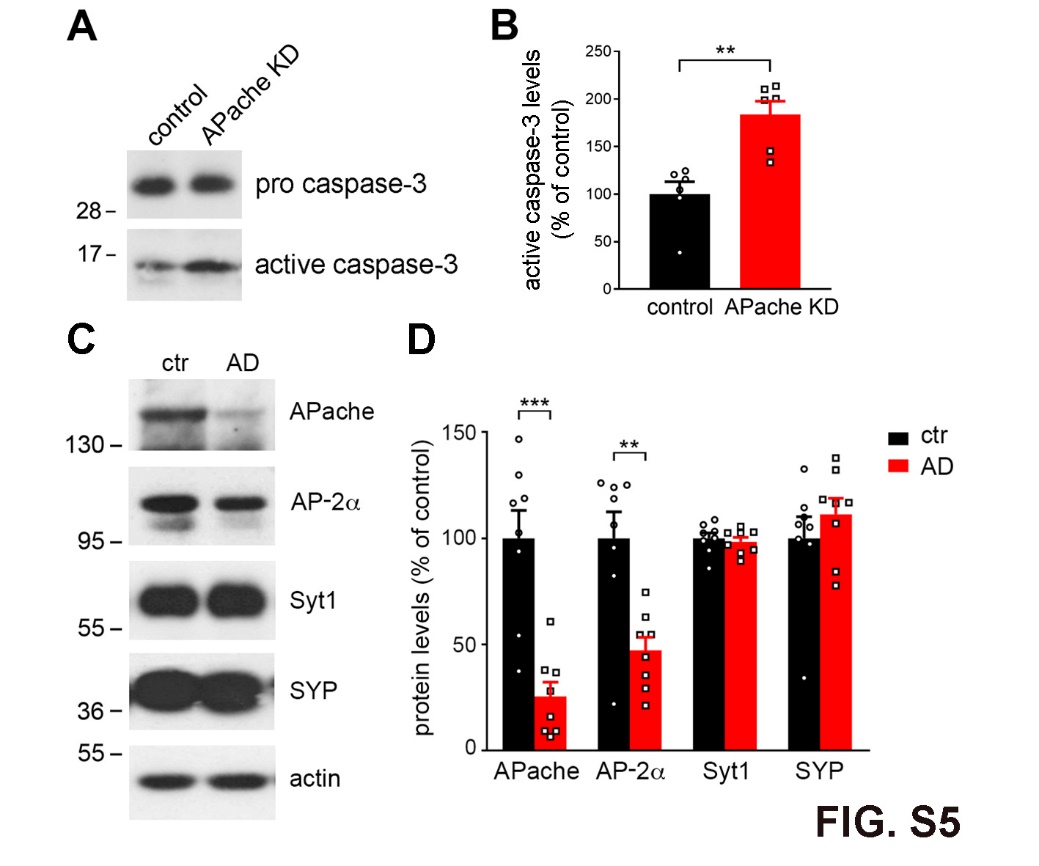


**Fig. S5 Decreased APache and AP-2α protein levels in human AD brains.**

**(A,B)** Representative blots **(A)** and quantitative analysis **(B)** of the expression level of the apoptotic marker active caspase-3 in lysates from cultured cortical APache KD neurons compared to controls at 17 DIV. Active caspase-3 protein levels were normalized to pro caspase-3 and expressed in APache KD neurons in percent of control neurons. (active caspase-3: 183.60 ± 14.26%, from n = 6 independent preparations). **(C,D)** Brain samples from late-onset sporadic AD patients (AD) and age-matched cognitively normally aging elderly subjects (ctr) were analyzed by Western blotting. Representative blots **(C)** and quantitative analysis of APache, AP-2α, synaptotagmin1 (Syt1) and synaptophysin (SYP) protein levels **(D)** are shown. AD patients presented drastically reduced expression levels of APache and AP-2α compared to controls, with preserved amounts of the SV proteins Syt1 and SYP. Immunoreactivity was normalized to Syt1 level. Protein levels in AD brains are expressed in percent of control brains. (APache AD: 25.52 ± 6.70%, AP-2α AD: 47.07 ± 6.33%, Syt1 AD: 98.40 ± 2.05%, SYP AD: 111.30 ± 7.47%, n = 8 control subjects and n = 8 AD patients). All graphs show means ± SEM. **p < 0.01; ***p < 0.001, unpaired Student’s *t*-test with Welch’s correction (B,D).


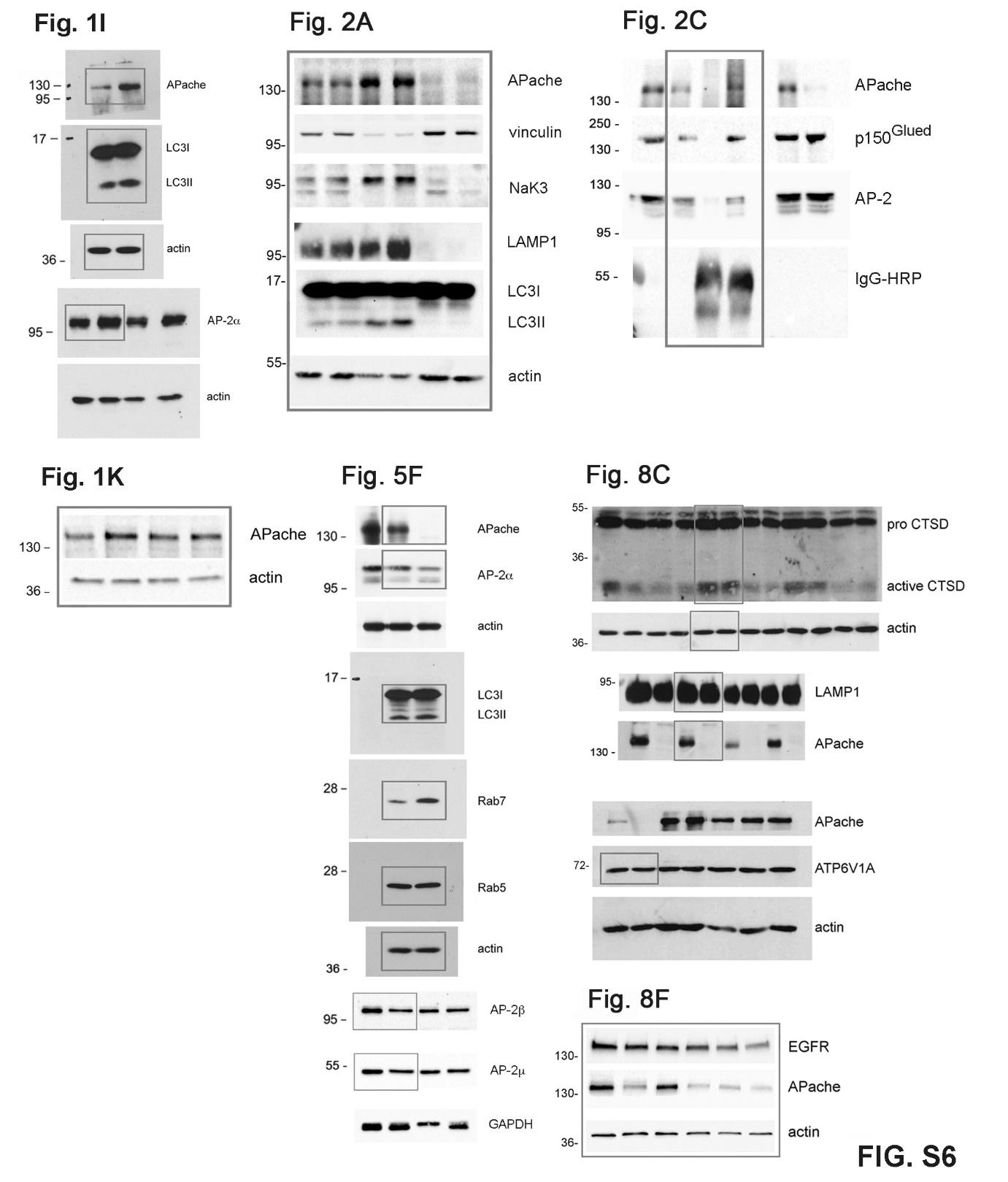


**Fig. S6 Full blots of Fig. 1I,K; Fig. 2A,C; Fig. 5F; Fig. 8C,F.**


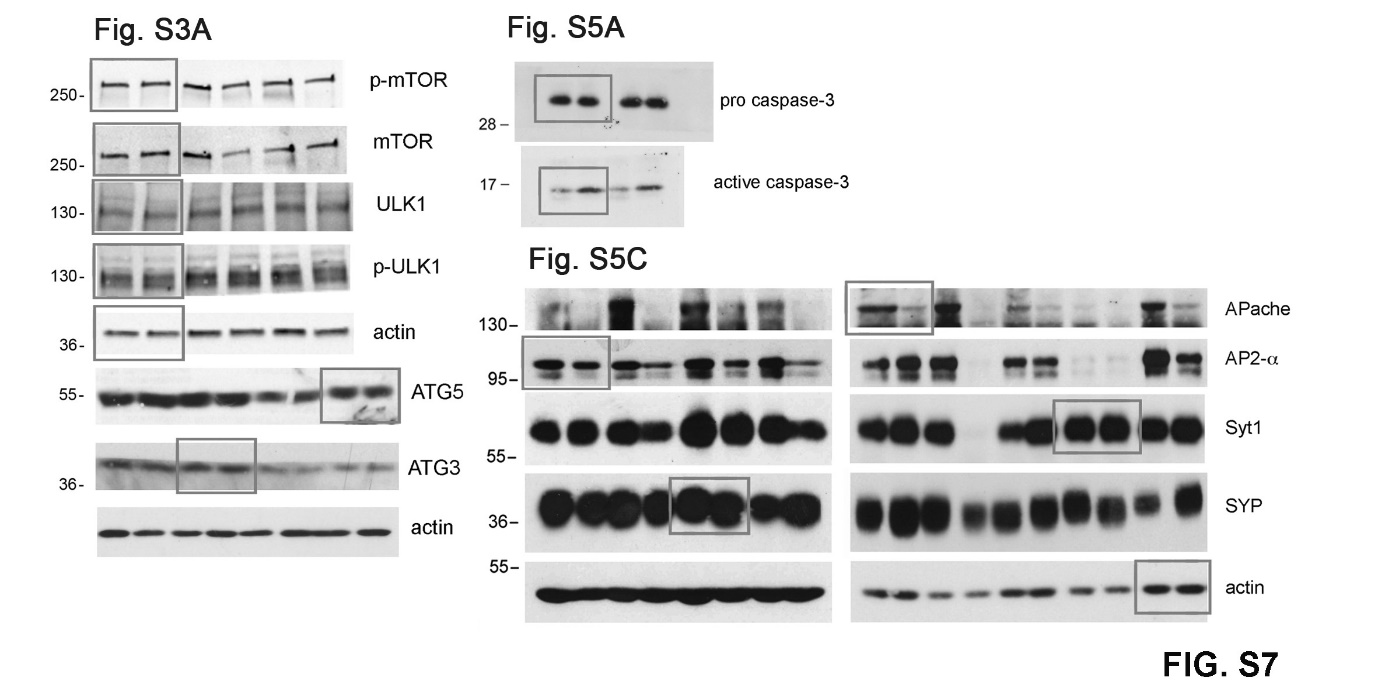


**Fig. S7 Full blots of Fig. S3A; Fig. S5A,C.**
